# Crisis and acute mental health care for people who have been given a diagnosis of a ‘personality disorder’: a systematic review

**DOI:** 10.1186/s12888-023-05119-7

**Published:** 2023-10-05

**Authors:** Lucy Maconick, Sarah Ikhtabi, Eva Broeckelmann, Alexandra Pitman, Kirsten Barnicot, Jo Billings, David Osborn, Sonia Johnson

**Affiliations:** 1 Division of Psychiatry, University College London, Maple House, 149 Tottenham Court Road, London, W1T 7BN UK; 2 Camden and Islington NHS Foundation Trust, St Pancras Hospital, 4 St Pancras Way, London, NW1 0PE UK; 3 Department of Health Services Research & Management, City, University of London, Northampton Square, London, EC1V 0HB UK

**Keywords:** Crisis care, Personality disorder, Complex emotional needs, Inpatient admission, Home treatment

## Abstract

**Background:**

People who have been given a diagnosis of a ‘personality disorder’ need access to good quality mental healthcare when in crisis, but the evidence underpinning crisis services for this group is limited. We synthesised quantitative studies reporting outcomes for people with a ‘personality disorder’ diagnosis using crisis and acute mental health services.

**Methods:**

We searched OVID Medline, PsycInfo, PsycExtra, Web of Science, HMIC, CINAHL Plus, Clinical Trials and Cochrane CENTRAL for randomised controlled trials (RCTs) and observational studies that reported at least one clinical or social outcome following use of crisis and acute care for people given a ‘personality disorder’ diagnosis. We performed a narrative synthesis of evidence for each model of care found.

**Results:**

We screened 16,953 records resulting in 35 studies included in the review. Studies were published between 1987–2022 and conducted in 13 countries. Six studies were RCTs, the remainder were non randomised controlled studies or cohort studies reporting change over time. Studies were found reporting outcomes for crisis teams, acute hospital admission, acute day units, brief admission, crisis-focused psychotherapies in a number of settings, Mother and Baby units, an early intervention service and joint crisis planning. The evidence for all models of care except brief admission and outpatient-based psychotherapies was assessed as low or very low certainty.

**Conclusion:**

The literature found was sparse and of low quality. There were no high-quality studies that investigated outcomes following use of crisis team or hospital admission for this group. Studies investigating crisis-focused psychological interventions showed potentially promising results.

**Supplementary Information:**

The online version contains supplementary material available at 10.1186/s12888-023-05119-7.

## Introduction

People with complex emotional needs (CEN) who may have been given a diagnosis of a ‘personality disorder’ require access to good quality services when they experience a crisis. For this population, crises can be a time of extreme emotional distress with increased risk of suicide, suicide attempts, non-suicidal self-harm (NSSH) and loss of social functioning. Some people with a ‘personality disorder’ diagnosis are frequent users of mental health crisis services [1] and can present to a spectrum of other services such as Emergency Departments and the police in a crisis [2]. A crisis can be difficult to define due its subjective and personal nature, but common themes in a range of definitions are that crises represent a time of heightened vulnerability, threaten the person’s sense of equilibrium, involve a period of overwhelming emotions and loss of control, can result in increased risk of self-harm or harm to others and/or might result in a person presenting to mental health or health services for urgent help [3]. Crisis and acute mental health services that a person may access include crisis or home treatment teams, acute psychiatric hospital admission, acute day units, crisis houses, crisis-focused psychological therapies or crisis houses/sanctuaries. The availability of different service models tends to vary considerably not only between countries, but between areas within the same country [4].

The term ‘personality disorder’ is controversial and historically the diagnosis has excluded people from services [5] as it was wrongly perceived as untreatable [6]. There is significant stigma associated with the label personality disorder, both amongst the wider public but also among clinicians in health services [7]. Some service user groups prefer the term ‘Complex Emotional Needs’ (CEN) and services in the United Kingdom are increasingly adopting this [8]. Some services use the term CEN to avoid the therapeutic pessimism and stigma associated with the diagnosis of “personality disorder”, often including in this term a broader population of people, including people who might have some needs associated with the diagnosis such as recurrent self-harm, but might not meet the full diagnostic criteria for a ‘personality disorder’[8]. However, there is presently no consensus about what terms and language should be used in the academic literature, mental health services and society at large to describe the difficulties that are currently labelled as a ‘personality disorder’. We advocate future work to develop less stigmatising and more valid ways of describing these difficulties. However, in this review we have used the ‘personality disorder’ term and construct to determine the inclusion criteria and search strategy, as this is the term used in the primary literature that we are reviewing.

Previous reviews examining crisis interventions and services for people with a ‘personality disorder’ diagnosis have limited inclusion criteria to randomised controlled trials (RCTs) or have not included studies reporting outcomes from commonly used crisis service models. A Cochrane review performed in 2022 of crisis interventions designed for people with a ‘personality disorder’ diagnosis found only two studies: a RCT of a brief admission intervention [9] and a pilot RCT of crisis planning [10, 11], both reporting no effect of the study intervention compared to treatment as usual. A systematic review conducted in May 2022 searched for randomised and observational studies of crisis-focused psychosocial interventions for ‘borderline personality disorder’ that can be delivered within acute care and found five studies [12]. In four of these studies the intervention was a form of psychological therapy and in one study the intervention was joint crisis planning. This review found that crisis psychosocial interventions are feasible but there was insufficient evidence to recommend a particular intervention. Both of these reviews focused only on crisis interventions designed specifically for people with a ‘personality disorder’ diagnosis, rather than more commonly delivered transdiagnostic models of crisis care. Neither review included studies that reported outcomes for people with a ‘personality disorder’ diagnosis following use of the crisis services that are currently available in the UK and many other countries, such as crisis teams, hospital admission, acute day services or crisis houses.

Other reviews have examined crisis interventions in a wider range of clinical other populations. For example, a 2015 Cochrane review of crisis interventions for people with severe mental illness (which included people with a ‘personality disorder’ diagnosis) did investigate the effects of crisis interventions, including models that a person with a ‘personality disorder’ diagnosis may be able to access such as crisis teams [13]. However, this review did not investigate effects or outcomes by diagnostic group. Given that it is thought that people with a ‘personality disorder’ diagnosis might experience particularly poor outcomes from crisis care and that recommendations are made in national guidelines to take particular care in avoiding over-use of inpatient crisis care for this diagnostic group [14], there is justification for investigating outcomes specifically for people with this diagnosis. A recent review of the qualitative literature has been performed by our research group synthesising experiences of non-inpatient crisis services, and found only a small number of published studies focusing on experiences of emergency departments [15].

Some commentators have argued that certain types of acute care, especially inpatient admissions, are thought to have potential to cause harm for people with a ‘personality disorder’ diagnosis, through encouraging regression, loss of control and coercion [11]. This is reflected in national guidelines for care of people with a ‘borderline personality disorder’ diagnosis, advising against admission to hospital in a crisis if possible, or advising that when admissions are used they are brief in duration [9, 16, 17]. These recommendations reflect the approach used in an evidence-based psychological intervention for people diagnosed with a ‘personality disorder’, Dialectical Behavioural Therapy (DBT), in which steps are taken to avoid admission to hospital where possible, with the aim of not reinforcing behaviours such as self-harm and to prevent the loss of coping skills [18]. However, clinical practice recommendations often cite a small number of studies relying on observational data [19] or expert opinion [16] and the strength of the evidence examining the benefits or harms of hospitalisation in this group appears to be low. Poor experiences of acute care may be due to a lack of the application of therapeutic models or interventions specifically aimed at supporting people with difficulties associated with a ‘personality disorder’ diagnosis in crisis services, and some suggest that a well-functioning treating team can provide an opportunity for useful care in acute inpatient settings [20]. Additionally, if it is the case that hospital admission is to be avoided, then there is little information about what alternative crisis care options might better meet the needs of this group. It is therefore important to establish what is known about the outcomes experienced following use of crisis care models for people with a ‘personality disorder’ diagnosis from existing evidence.

No previous review has focused on the outcomes experienced by people with a ‘personality disorder’ diagnosis following use of the currently available models of crisis and acute care. The inclusion criteria for the current review have been kept broad given that this field of research is in its early stages. It is therefore of benefit to describe all preliminary data that is available. This will add to the previous more focused reviews which have only found a small handful of eligible studies.

## Aims

We aimed to summarise current knowledge of the clinical and social outcomes experienced by people with a ‘personality disorder’ diagnosis following use of acute/crisis services and interventions. We also aimed, subject to sufficient evidence being available, to compare the effectiveness of the different available models of crisis care for people with a diagnosis of a ‘personality disorder’.

## Methods

We performed a systematic review following PRISMA guidelines [21]. The protocol was pre-registered on Prospero prior to commencement of screening (PROSPERO 2022 CRD42022313720).

### Study inclusion criteria

Inclusion criteria were peer-reviewed published studies in which individuals studied were adults (> 16 years), had a diagnosis of a ‘personality disorder’ or were identified as having CEN, had used at least one type of crisis or acute care service and for whom at least one clinical or social outcome measure was reported following use of crisis care.

Study types eligible for inclusion were quantitative study designs, including RCTs, quasi-experimental studies and observational studies reporting pre-post outcomes. Qualitative studies, case control studies, case reports and opinion pieces were excluded. Reviews were not included but the reference lists searched for eligible studies.

#### Population

Studies were included if the majority of adults studied (> 50%) had been given a diagnosis of a ‘personality disorder’. Additionally, studies in which < 50% participants were given a diagnosis of a ‘personality disorder’ were included if there were outcomes reported separately for a subgroup of people with a ‘personality disorder’ diagnosis.

A specific research diagnosis (for example a ‘personality disorder’ diagnosis as recorded by the Millon Clinical Multiaxial Inventory) was not specified in order to keep the inclusion criteria broad and therefore be able to describe all available literature in this field.

Articles that studied individuals presenting with self-harm without other features of a ‘personality disorder’ diagnosis were not included, but given that authors may be reluctant to use the term ‘personality disorder’, papers referencing recurrent self-harm and/or emotional dysregulation in the abstract were taken to the full text screening stage. Studies were included if authors stated that they were using an alternative less stigmatising term for ‘personality disorder’ such as complex emotional needs but that > 50% of participants would still meet the criteria for the diagnosis or outcomes were reported separately for a subgroup with the diagnosis.

#### Intervention

Crisis or acute care refers to mental health services that provide urgent support for mental health. Studies were included if the authors described the service studied as a ‘crisis’ or’acute’ service. If authors did not state if the service was a crisis or acute service, studies were included if: 1) service duration was < 3 months and 2) participants could be urgently referred to the service directly from emergency departments or following presentation in crisis or 3) if the service was considered an alternative to an acute psychiatric hospital admission. Types of services that were anticipated to be included were: crisis resolution (or home treatment) teams, crisis houses/sanctuaries, crisis cafes, acute inpatient hospital admission, acute day units, acute Mother and Baby units, and services offering crisis-focused psychosocial or psychological interventions. Crisis services that provide an assessment function in a one-off contact but that do not provide ongoing crisis care, (such as crisis lines or psychiatric liaison teams in emergency departments providing an assessment but no ongoing treatment) were excluded. We excluded planned hospital admissions for psychotherapy (whether brief or longer term), longer term treatment programs such as day hospitals that provide planned admissions and rehabilitation, and admissions to substance misuse rehabilitation or detoxification units. Studies evaluating the effectiveness of medications in acute crisis were not included.

#### Comparator

Studies were eligible if they reported comparison of outcomes between different crisis care models or between a crisis care model and treatment as usual. However, studies without a comparator were also included.

#### Outcomes

Studies were included that provided an outcome measure from at least one type of crisis care for this patient group, measured at least once within 1 year of discharge from the crisis service. This included pre-post outcomes and post-intervention outcome comparisons. Outcomes were expected to fall within categories of symptomatic improvement, service use, adverse events (such as self-harm or suicide), and social functioning, but we did not prespecify which outcomes would be included.

No limits were placed on language of the manuscript at the search stage.

### Search strategy and information sources

We searched eight electronic databases on the 2nd March 2022: OVID Medline (for articles published between 1946 and the 1^st^ March 2022), Embase (1974 to 2022 Week 08), OVID PsycInfo (1806 to February week 08 2022), OVID PsycExtra (1908 to March 1st 2022), Web of science (1900 until February 24^th^ 2022), HMIC via OVID (1979 and March 2022), CINAHL Plus via Ebschohost (1976 to 25^th^ February 2022), Clinical Trials (Year 2000 to 24^th^ February 2022) and Cochrane CENTRAL (until March 2022). OVID PsycExtra and CINAHL provide grey literature records.

Databases were searched using Medical Subject Heading (MeSH) and keywords for: “complex emotional needs”, personality disorders, emotional dysregulation AND crisis services (for full details of search terms please see Supplementary material 1). The search strategy was developed with the input of a specialist librarian and discussed with the project Lived Experience Working Group. No limits on year of publication or language were set. Reference lists of review articles found were searched for eligible studies. In addition, backward citation searching was performed by manually searching reference lists of eligible studies. A forward citation search was performed for articles that cited eligible studies using Scopus.

### Data extraction and synthesis

One reviewer screened all titles and abstracts of the studies identified (LM), with a second reviewer independently screening 10% of citations (SI). Conflicts were resolved by discussion and where necessary in consultation with a third reviewer (SJ). Once titles and abstracts that did not meet the inclusion criteria were excluded, full texts of articles were screened by one reviewer (LM), and 10% of full texts were independently screened by a second reviewer (SI). Screening was managed in Rayyan software for systematic reviews [22]. Data from studies in languages other than English were extracted using Google document translate, with translations back-translated into English to ensure that the meaning was correctly interpreted. Included studies in languages other than English were also read by a native speaker of the language within the research group who checked the quality assessments.

We extracted data on the publication year, study design, sample, setting, population, intervention studied, outcomes and outcome measurement timepoints into a standardised form that had been piloted using a subset of articles.

We grouped studies according to those describing similar models of care and then sub-grouped by the outcome reported. We assessed whether there were multiple studies reporting outcomes from the same models of care and using similar outcome measures, and therefore whether it would be possible to perform a meta-analysis. The very small number of studies that did report similar outcomes for the same models of care did not use comparable measures (see range of measures used in Table 2) and so it was not possible to perform a meaningful meta-analysis. As there was some variation in the descriptions of similar models of care within groups we performed a narrative synthesis. The variation in services included in each model of care category is described for each group in the results.

### Quality assessment and certainty of evidence scoring using GRADE

To assess individual study quality we used the ROBINS-I tool for observational studies [23], the Cochrane risk of bias tool (RoB 2) for RCTs [24] (with the cluster adaptation for cluster RCTs) and the Newcastle Ottawa Scale [25] for observational studies reporting only change in pre and post intervention outcomes over time. Independent quality assessment was conducted by a second reviewer (SI) for 15% of all studies. Quality assessments in languages other than English were also checked by researchers who are native speakers of the language. Examples of quality assessments performed using the RoB 2 and ROBINS-I tool are included in Supplementary material 4 (S4).

We assessed the certainty of the evidence available using the Grading of Recommendations, Assessment, Development and Evaluations (GRADE) scoring system [26]. GRADE criteria were adapted for a narrative synthesis approach using methods described by Murad et al. [27]. A GRADE score was produced for each group of outcomes for each model of care. The GRADE scoring system rates the evidence in terms of five domains: study quality, inconsistency, indirectness, imprecision and publication bias, with concerns in each domain resulting in a downgrading in the certainty of evidence. Further details of the criteria used for assessing GRADE scoring for each model of care is provided in Supplementary material 3 (S3).

## Results

**Fig. 1:**
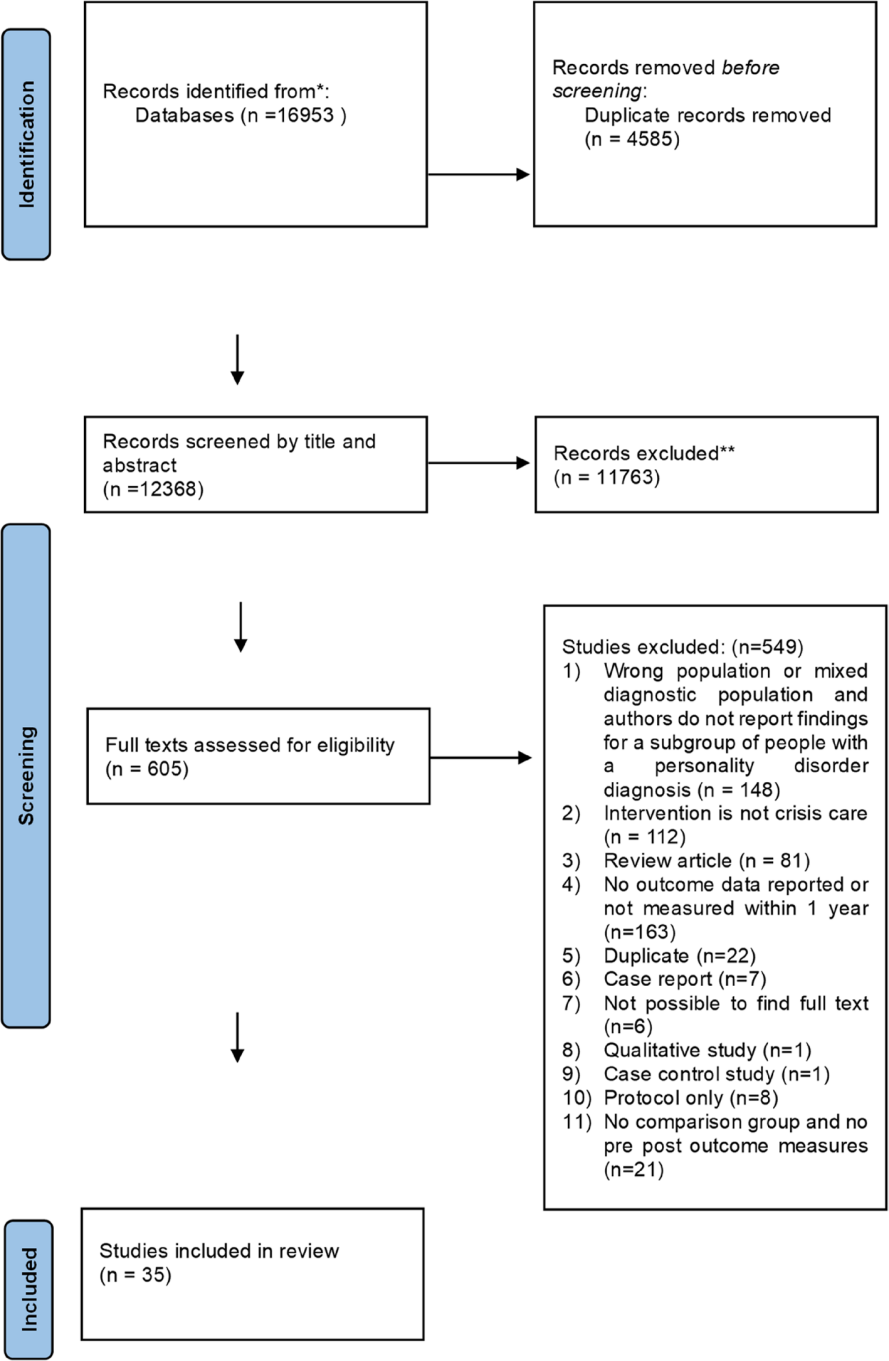
PRISMA diagram

Searches of bibliographic databases (including grey literature databases) returned 16953 results, of which 4585 were duplicates. We screened 12368 titles and abstracts, with 11763 results excluded. We assessed 605 full texts for eligibility resulting in 35 studies included in the review. Reference lists of reviews that had been identified were searched and forward and backward citation searching was performed but this did not identify any new studies meeting inclusion criteria (see Fig. 1, PRISMA flow chart). No grey literature that met the inclusion criteria was found.

Based on the 10% of records abstract screened by a second reviewer, there was initially 85% agreement. After discussion all discrepancies were resolved. For full text screening, there was 83% agreement. Discrepancies were resolved after discussion, with two discrepancies resolved by a third reviewer. At both stages there was revisiting of the inclusion criteria with both reviewers.

### Study characteristics

The 35 eligible studies included were published between 1987 and 2022, with 21 studies published since 2010. See Table 1 for details of all included studies. Studies were conducted in the UK (*n* = 4),

**Table 1:** Characteristics of included studies

Authors	Sample with ‘personality disorder’ diagnosis	Population	Subgroup with PD diagnosis Y/N	Subtype of ‘personality disorder’	Age (mean)	Gender % female	Ethnicity or Country of Birth		Country	Study design	Intervention	Intervention duration	Control/Comparison
Andreoli 2016 [28]	170	Participants all met the DSM-IV criteria for BPD and MDD, presenting to ER after suicide attempt or self-harm	N	BPD	31.9	84.1		NR	Switzerland	RCT	Abandonment psychotherapy	3 months	TAU
Barbato 2011 [29]	36	All patients admitted or discharged from 262 General Hospital Psychiatric Units in May 2004 during 12 day index period	Y	All subtypes	41.2^a^	43.2^a^		NR	Italy	Cohort study with pre post outcomes reporting change over time	Brief admission	Up to 12 days	NA
Berrino A 2011 [30]	200	Adults (18–65) referred to the emergency department of large teaching hospital who met DSM-IV criteria for BPD + presence of severe deliberate self harm	N	BPD	Intervention: 32.6 Control: 31.5	Intervention: 87, Control: 83		NR	Switzerland	Non randomised controlled study with historical controls	Crisis intervention in the general hospital	Mean 4.6 days	Participants presenting to same service environment before crisis hospitalisation was implemented
Booth 2014 [31]	70	Adults must be over 18 yrs, hospital inpatients, have a history of NSSI or strong ideation, study welcomed people with BPD. Final sample 61.4% people with BPD	Total sample > 50%	BPD	35.22	80.7		NR	Ireland	Cohort study with pre post outcomes reporting change over time	DBT skills group	NR	NA
Borschmann 2013 [11]	88	Adults accessing community mental health teams in south east london who met diagnostic criteria for borderline personality disorder, had self harmed in the previous 12 months, were under the care of a CMHT and did not have a psychotic illness	N	BPD	35.8	83		1.1% Asian, 10.2% Black 73.9% White 8.0% white 6.8% other	UK	Pilot RCT	Joint crisis planning	1 week	TAU
Branjerdporn 2021 [32]	25	Young adults (age 18 to 25 years) admitted to a specialised acute psychiatric unit for young adults	Y	All subtypes	20.63	20		Country of birth: 7% outside Australia 1st nation status 0%	Australia	Cohort study with pre post outcomes reporting change over time	Acute admission to Young Adult mental health unit	Mean 13.44 days	NA
Breslow 1993 [33]	69	Adults admitted to crisis beds at a psychiatric emergency service- those judged as likely to significantly improve within a 72 h timeframe	Total sample > 50% BPD	BPD	Study 1: 36.3 Study 2: 35.6^a^	Study 1: 45% Study 2: 49%^a^		NR	USA	Cohort study with pre post outcomes reporting change over time, replicated 1 year apart	Crisis beds	3 days	NA
Damsa 2003 [34]	507	Adults > 16 yrs presenting to ER in crisis	Y	All subtypes	Pre: hospitalised 45, OP 41 Post: Hospitalised: 48 OP 41^a^	NR		NR	Luxembourg	Non randomised controlled study with historical controls	Crisis intervention in psychiatric emergencies service	Up to a few weeks	Use of psychiatric emergency service before crisis intervention introduced
Damsa 2005 [35]	190	Compared adults (> 16yrs) who consulted the psychiatric emergency department over a period of 8 months in 2002 with those who consulted over the same period in 2003 after crisis intervention was implemented	Y	BPD	Control group: Hospitalised mean age 48.7, Not hospitalised 44.4, Intervention group: hospitalised 49.3, not hospitalised 43.9^a^	56^a^		NR	Luxembourg	Non randomised controlled study with historical controls	Crisis intervention in psychiatric emergencies service	Up to a few weeks	Use of psychiatric emergency service before crisis intervention introduced
Eckerstrom 2022 [36]	63	Patients of 2 OP units and 2 psychiatric hospital wards in Stockholm specialised in BPD and anxiety disorders. Participants needed to have a clinical history and current symptoms of emotional instability and a history of self-harm, and at least one previous period of inpatient care	Total sample > 50% BPD	BPD	33.7^a^	78^a^		NR	Sweden	Cohort study with pre post outcomes reporting change over time	Patient-initiated brief admission	1–3 days, max 3 uses per month	NA
Gebhardt 2016 [37]	18	Inpatients of a general psychiatric hospital	Y	Mixed (8), Dependent (3), EUPD (2), histrionic (2), narcissistic (2), NOS (1)	48.3	66		NR	Germany	Cohort study with pre post outcomes reporting change over time	Acute psychiatric hospitalisation	Mean 1.4 months	NA
Giese 1990 [38]	9	Patients aged 18–45 yrs admitted to a psychiatric ward over a 3 month period. Must meet DSM-II criteria for axis II diagnosis and have an axis I diagnosis of an affective disorder	Y (and total sample > 50% BPD)	BPD	30^a^	88^a^		NR	USA	Cohort study with pre post outcomes reporting change over time	Acute psychiatric hospitalisation	Mean 22.8 days	NA
Grenyer 2018 [39]	642	Patients presenting to the hospital inpatient unit or emergency department: aged over 12 yrs, at least one inpatient admission during baseline 18 months, had a primary diagnosis of a personality disorder based on ICD10	N	All subtypes	36.85	Intervention group: 46 Treatment group: 55.4		NR	Australia	Cluster RCT	Brief crisis focused psychological intervention	One month of weekly contact	Usual medical care and community treatment by health care professionals, waitlist for psychological treatments
Huxley 2019 [40]	67	Aged > 18yrs, presenting with suicidal thoughts or plans, recent episodes of self harm behaviour, emotional dysregulation and/or personality disorder	N	All subtypes	31.54	75		NR	Australia	Cohort study with pre post outcomes reporting change over time	Brief crisis focused psychological intervention	1 month	NA
Koekkoek 2010 [41]	11	Adults 18–60 yrs, received treatment in a day hospital with a formal DSM-IV diagnosis of BPD and history of repeated or long-term admissions	N	BPD	43.55	100		NR	Netherlands	Cohort study with pre post outcomes reporting change over time	Preventative brief psychiatric admissions	Variable length of stay over 6 months	
Laddis 2010 [42]	58	Consecutive admissions to a crisis stabilisation unit with a diagnosis of BPD or CPTSD	N	BPD	Control group 33.2 experimental group 37.2	Control group 96 Intervention group 75		NR	USA	Non randomised controlled trial with contemporaneous controls	Psychotherapeutic crisis intervention according to the Cape Cod Model	2–3 days	TAU- medication, supportive psychotherapy, problem solving, occasional analysis of the transference and elements of DBT
Lariviere 2010 [43]	20	Adults > 18 yrs who were treated in a psychiatric day hospital and were diagnosed as having 1) psychotic disorders, 2) mood and anxiety disorders 3) cluster B personality disorders	Y	Cluster B personality disorders	38.4	90		100% Caucasian	Canada	Cohort study with pre post outcomes reporting change over time	Acute day hospital	mean 8 weeks	NA
McQuillan 2005 [44]	87	Patients whose main problems are recent suicidal or parasuicidal behaviour, severe impulsive disorders, anger problems or multiple therapeutic failures. 92% of participants screened positive for BPD	N	BPD	37	81		NR	Switzerland	Cohort study with pre post outcomes reporting change over time	3 week intensive DBT program aimed at crisis support, with optional initial stay in crisis centre of up to 2 nights	3 weeks	
Mellsop 1987 [45]	57	Adults (aged 20–60 yrs) admitted to a general hospital psychiatric unit over a 12 month period	Y	All subtypes	35^a^	58		NR	New Zealand	Cohort study with pre post outcomes reporting change over time	Acute psychiatric hospitalisation	NR	NA
Nehls 1994 [46]	5	Clients of a designated community mental health centre, meeting the DSM-III criteria for BPD and involved in brief hospital treatment program for at least 1 year	N	BPD	NR	NR		NR	USA	Cohort study with pre post outcomes reporting change over time	Brief hospital treatment plan	Brief admissions 48–72 h	NA
Pavan 2003 [47]	22	Young adults in emotional crisis. Referrals received from GPs, emergency room psychiatrists, community mental health facilities or self-referrals	Y (and total sample > 50% PD)	Depressive (20.9%), OPD (18.6%) BPD (16.3%), avoidant (9.3%), dependent (9.3%) and schizotypal (9.3%)	30.9^a^	76		NR	Italy	Cohort study with pre post outcomes reporting change over time	Crisis psychotherapy	10 weeks	NA
Savard 2019 [48]	270	260 patients with a DSM-IV personality disorder diagnosis who were experiencing a crisis episode, and who completed the day hospital treatment program	N	68% BPD, Cluster B 14%, narcissistic 7.4%, mixed 5.8%, dependent and histrionic (1.6%) OCPD (1.2%), schizotypal (0.4%)	18 to 24 years = 23.8%, 25–30 years = 22.7%, 31 to 40 years = 26.2%, 41 to 50 years = 17.7% and 51 + years = 9.6%	78^a^		NR	Canada	Cohort study with pre post outcomes reporting change over time	Time limited day hospital crisis treatment for personality disorders	6 weeks	NA
Shergill 1997 [49]	8	All admissions to an acute day unit over a one year period	Y	All subtypes	37.3	54^a^		NR	UK	Cohort study with pre post outcomes reporting change over time	Acute day hospital	Mean 157.5 days	NA
Springer 1995 [50]	31	Adults admitted to a general inpatient psychiatry ward at a university hospital who met criteria for a diagnosis of a personality disorder diagnosis using the MCMI-II	N	BPD	31.4	68		87.1% White 6.5% African American 3.2% Hispanic 3.2% Asian	USA	RCT	Creative coping' skills training groups on short term inpatient ward	Mean 2 weeks	Wellness and lifestyles groups- discuss items of interest to patients but not in a psychotherapeutic manner
Turhan 2016 [51]	27	All patients who had primary ICD10 diagnosis of BPD taken on by the Edinburgh Intensive Home treatment team between 2010 and 2013	N	BPD	39 (median)	100		NR	UK (Scotland)	Cohort study with pre post outcomes reporting change over time	Intensive home treatment team	NR	NA
Tyrer 1994 [52]	50	Adults (aged 16-65yrs) presenting in psychiatric emergeny at a general hospital over a 14 month period.. Subgroup analysed with a personality disorder diagnosis based on ICD classification	Y	All subtypes	EIS group: 35 HS group: 30 (median)^a^	55		Caucasian 63% Other 25% (PD group only)	UK	RCT	Early intervention service	12 weeks	Conventional hospital based psychiatric services
Uhlmann 2008 [53]	63	All admissions in the area with an acute crisis and the main diagnosis of F6 or F4 according to ICD10 without a comorbid schizophrenic disorder. If the main diagnosis was F4 then a personality disorder had to be present as a secondary diagnosis. Acute crises had to involve suicidal or violent behaviour	N	All subtypes	Before group: 32.6 After group: 31.5	Before: 55 After: 78		NR	Germany	Non randomised controlled study with historical controls	Specialised hospital admission ward for patients with personality disorder diagnoses in acute crisis	Before group: mean 32.5 days After group 43 days	Admission to general acute psychiatric ward without a disorder specific psychotherapeutic ward concept
Unger 2013 [54]	68	Adults (> 18 yrs) recruited from psychiatric unit in a clinic in Berlin with a major depressive episode or recurrent depression as the principal diagnosis. Analysed a subgroup with comorbid personality disorder diagnosis	Y	OCPD (13.1%), avoidant (12.5%), BPD (9.5%), depressive 9.5%), narcissistic (5.4%), dependent (4.2%), paranoid (3.6%), histrionic (3%), schizoid (1.2%), antisocial (1.2%), passive-aggressive (1.2%), schizotypal (0.6%)	48.17	57.4		NR	Germany	Cohort study with pre post outcomes reporting change over time	Acute psychiatric hospitalisation for depression	Mean 60.31 days	NA
Van Kessel 2002 [55]	21	Clients of an inpatient mental health facility who had a DSM-IV diagnosis of borderline personality disorder	N	BPD	30	100		BPA group: 90% Caucasian, 10% Pacific Island Control group: 64% Caucasian 27% pacific islander 9% other	New Zealand	Non randomised controlled trial with matched contemporaneous controls	Brief planned admission	NR	Standard care through community and inpatient services
Vazquez-Bourgon 2021 [56]	27	Adults (> 18 yrs) admitted to an acute psychiatric day hospital between January 2015 and January 2017. Inclusion criteria: 1) main DSM-IV diagnosis of non affective psychotic disorder, bipolar disorder, mood and anxiety disorder, personality disorder 2) present acute symptomatology for which the patient needed acute intensive treatment 3) voluntarily admitted to the day hospital	Y	All subtypes	37.3	74		NR	Spain	Cohort study with pre post outcomes reporting change over time	Acute day hospital	20.0 days	NA
Westling 2019 [9]	125	Adults (18–60 yrs) attending 4 psychiatric inpatient services with a current episodes of self harm and recurrent suicidal behaviour, 3 or more diagnostic criteria for BPD, 7 or more days of hospital admission or presenting to ER 3 or more times in the last 6 months. 63.4% had diagnosis of BPD and 19.2% had diagnosis of other personality disorder	N	BPD	32	85		NR	Sweden	RCT	Brief admission	Maximum 3 nights	TAU, including general psychiatric admission
Wright 2020 [57]	18	All English speaking mother infant pairs who had an inpatient stay of 4 or more days in a 3-bed MBU. Personality disorder was a comorbid diagnosis	Y	All subtypes	32.2	100		64% NZ European	New Zealand	Cohort study with pre post outcomes reporting change over time	Mother and baby unit	Approximately 3 weeks	NA
Yen 2009 [58]	50	Participants recruited consecutively from a 5 day DBT partial hospitalisation program for women. Participants had to be between 18 and 65 years of age and meet full criteria for BPD. Exclusion: schizophrenia, bipolar, cyclothymic disorder, substance dependence or mental retardation	N	BPD	NR	100		NR	USA	Cohort study with pre post outcomes reporting change over time	5 day DBT partial hospitalisation program	5 days	NA
Yoshimatsu 2015 [59]	64	A sample of 245 psychiatric inpatients divided into 2 groups based on whether they screened positive on the Mclean screening instrument for BPD	Y	BPD	38	73		92.2% White	USA	Cohort study with pre post outcomes reporting change over time	Acute psychiatric hospitalisation in a mood disorders unit	6.5 days	NA
Zimmerman 2022 [60]	182	Diagnosis of BPD. To take part in the virtual program they were required to have a computer, tablet or smartphone with access to the internet (those who did not have these had a kindle tablet and internet provided short term). Excluded if had a primary substance use disorder or imminent suicidal or homicidal ideation with plan and intent	N	BPD	In person group: 33.82, Telehealth: 34.86	In person group: 74.4 Telehealth: 73.4		In person group: 62.4% White 17.1% Hispanic 6.8% Black 3.4% Asian 9.4% other Telehealth group: 73.4% White 10.9% Hispanic 12.5% black 0% Asian 3.1% other	USA	Non randomised controlled study with historical controls	In person partial hospitalisation program	Mean 13.2 days in telehealth group 9.6 days in in person group	Telehealth version of the program

*BPA* Brief planned admission, *BPD borderline personality disorder, CMHT* Community mental health team, *DBT* Dialectical Behavioural Therapy*, DSM* Diagnostic and Statistical Manual*, ER* Emergency room, *EUPD* Emotionally Unstable Personality Disorder*, MB* Mother and Baby Unit*, MDD* Major Depressive Disorder*, MCM-II* Millon Clinical Multiaxial Inventory*, NOS* Not otherwise specified, *PD* 'Personality disorder', *RCT* Randomised controlled trial, *NA* Not applicable, NR Not reported, *NSSI* Non suicidal self injury*, NZ* New Zealand*, TAU* Treatment As Usual

^a^ Authors reported population characteristics only for total sample rather than a subgroup of those with a personality disorder diagnosis

Australia (*n* = 3), Switzerland (*n* = 3), USA (*n* = 8), Sweden (*n* = 2), Germany (*n* = 3), Italy (*n* = 2), New Zealand (*n* = 3), Canada (*n* = 2), Luxembourg (*n* = 2), Spain (*n* = 1), Netherlands (*n* = 1) and Ireland (*n* = 1). The majority of studies were published in English, with two studies published in French [35, 61] and one study published in German [53].

For 22 studies the whole or > 50% of the included population had a diagnosis of a ‘personality disorder’, whereas for 13 studies people with this diagnosis were a subgroup. For 19 studies, participants were those diagnosed with a ‘borderline personality disorder’ diagnosis, one study included only those with a Cluster B ‘personality disorder’ diagnosis subtype and the remaining 15 studies included participants with a mixture of the subtypes of the ‘personality disorder’ diagnoses (see Table 1). There were four studies in which the term ‘personality disorder’ was not included in the title and abstract, but when the full text was read it was found that > 50% of participants met the criteria for a ‘personality disorder’ diagnosis [9, 31, 36, 47]. These studies used terms such as ‘individuals with emotional instability and self-harm’[36] and ‘adults who self-harm and who are at risk of suicide’[9] to describe the population studied in the title and abstract. One study included participants with a ‘borderline personality disorder’ diagnosis and complex post-traumatic stress disorder (CPTSD) together as ‘complex post traumatic syndromes’[42]. Sample sizes for people with a diagnosis of a ‘personality disorder’ ranged from 5 to 642 individuals. Eight studies only reported demographic characteristics of the total sample, not the subgroup of people with a ‘personality disorder’ diagnosis (indicated by a * in Table 1). In 21 studies > 60% of participants were female. Only nine studies reported the ethnicity or country of birth of participants, which was predominantly White in all studies.

Study types were predominantly non-randomised designs: five RCTs, one cluster RCT, two non-randomised studies with contemporaneous controls, six non-randomised studies with historical controls and 21 cohort studies reporting only changes in pre-post outcome measures over time.

The models of crisis care that were evaluated were: crisis teams (*n* = 1 study), acute psychiatric hospital admissions (*n* = 7 studies), a brief admission model (*n* = 6 studies), acute day units/hospitals (*n* = 6 studies), psychosocial crisis interventions based in general hospitals or psychiatric emergency services (*n* = 5 studies), crisis focused psychotherapies based in outpatient settings (*n *= 5 studies), DBT based groups delivered on acute inpatient wards (*n* = 2 studies), Mother and Baby units (*n* = 1 study), a community early intervention service (*n* = 1 study) and joint crisis planning (*n* = 1 study). The study of the early intervention service pre-dated the development of the current psychosis-focused model of early intervention. Brief admission referred to a distinct model of care from general acute psychiatric hospital admission, designed specifically for people with a ‘borderline personality disorder’ diagnosis [62]: in this model of care the duration of hospitalisations (and in some cases the frequency) are agreed in advance of the admission between service user and treating team and once in hospital a shared risk management plan is followed. In one study brief admissions were also used preventatively [41].

Where a comparison group was used, the intervention was generally 'treatment as usual’ (TAU). ‘Treatment as usual' most commonly involved a combination of intensive community treatment and use of hospital admission to manage risk if needed. Interventions most commonly maintained access the treatment as usual in addition to the intervention to manage risk. For further information on comparison groups please see Table 1.

The tools used to measure clinical and social outcomes across studies varied widely (Table 2). No outcome measures were found that appeared to have been developed specifically for the population of a people with a ‘personality disorder’ diagnosis, other than potentially the ‘distress tolerance’ scale [31, 63].

**Table 2:** Outcomes reported across studies

Outcome	Measurement tools	Number of studies using measurement tool
Suicide/suicide attempt/suicidal ideation	Re-presentation to services with suicidal ideation or attempt	2 studies [28, 30]
	Time to repeat suicide attempt	2 studies [28, 30]
	Suicidal ideation on ASIQ	1 study [50]
Self-harm/NSSI	Mean number of reported NSSI events within 2 weeks, 6 weeks or 6 months	3 studies [9, 11, 31]
	% who had self-harmed over 6 months	1 study [11]
Symptomatic improvement	BDI	6 studies [38, 40, 44, 47, 54, 58]
	HRSD	2 studies [38, 54]
	HAS	1 study [38]
	GAS	2 studies [38, 47]
	SCL-90-R	2 studies [38, 43]
	BSI	4 studies [42, 53, 54, 58]
	CGI	4 studies [37, 51, 54, 56]
	GAF	2 studies [37, 54]
	OQ-45.2	1 study [48]
	PSAS	1 study [33]
	CPRS	1 study [52]
	MADRS	1 study [52]
	BAS	1 study [52]
	WAI-C	1 study [11]
	WAI-T	1 study [11]
	WEMWBS	1 study [11]
	Modified remission from depression questionnaire	1 study [46]
	HADS-D	1 study [11]
	HADS-A	1 study [11]
	Perceived distress	1 study [43]
	Beck Hopelessness Scale	2 studies [44, 58]
	Hopelessness Scale	1 study [50]
	Dissociative Experiences Scale	1 study [58]
	STAXI	3 studies [47, 50, 58]
	STAI	1 study [47]
	BPRS	3 studies [29, 42, 56]
Health and social functioning	Health of the Nation Outcome Score	2 studies [32, 56]
Satisfaction with services/experience with services	CUPPS questionnaire	1 study [60]
	TES	1 study [11]
	CSQ	1 study [11]
Hospital admission or readmission	Mean days in hospital over 6, 12 or 18 months	5 studies [9, 30, 39, 41, 55]
	Time to readmission or hospitalisation	2 studies [28, 30]
	Mean number of inpatient admissions over 18 months	1 study [39]
	Rate of hospitalisation	2 studies [34, 35]
Health related quality of Life	EQ-5D	1 study [36]
Therapeutic alliance	Clinician rated agreement with treatment on a Likert Scale	1 study [41]
Social functioning	Life Habits Scale	1 study [43]
	Satisfaction with Social Participation	1 study [43]
	WSAS	1 study [11]
	Social Adaptation Self Evaluation Scale (SASS**]**	2 studies [44, 47]
Distress tolerance	Distress Tolerance Scale	1 study [31]
Coping skills	CCQ Creative Coping Questionnaire	1 study [50]
Mother infant relationship	CARE index	1 study [57]
Adaptive functioning	SAS-M	1 study [45]
	GHQ-9	1 study [45]
	CGHQ	1 study [45]

*HAS* Hamilton Rating Scale for Anxiety, *GAS* Global Assessment Scale, *SCL-90R* Revised Symptom Checklist, *BPRS* Brief Psychiatric rating scale. *HRSD* Hamilton Rating Scale for Depression, *GAF* Global Assessment of Functioning, *CGI* Clinical Global Impression Scale, *BSI-GSI* Brief Symptom Inventory, *HoNOS* Health of the nation outcome score, *GHQ-9* General Health Questionnaire SAS-M Social Adjustment Scale-Modified CGHQ- clinical general health questionnaire. *EQ-5D* EuroQol 5 dimensions. *NSSI* Non-suicidal self injury. *OQ-45.2* Outcome Questionnaire, *SCL-90R* Revised Symptom Checklist, *BDI* Beck Depression Inventory, *BSI* Brief Symptom Inventory, *STAXI* State-Trait Anger Expression Inventory. *BHS* Beck Hopelessness Scale, *SASS* Social Adaption Self-Evaluation Scale, *QoL* Quality of Life, *GAS* Global Assessment Scale. *PSAS* Psychiatric Symptom Assessment Scale, *ASIQ* Adult Suicidal Ideation Questionnaire, *CCQ* Creative Coping Questionnaire, *GAF* Global Assessment of Function, *CARE* Child and Adult Relational Experimental Index, *CPRS* Comprehensive Psychopathological Rating Scale, *MADRS* Montgomery and Asberg Depression Rating Scale, *BAS* Brief Scale for Anxiety HADS-A. *WAI* Working Alliance Inventory *CSQ* Client Satisfaction Questionnaire *SES* Social Engagement Scale, *WEMWBS* Warwick-Edinburgh Mental Wellbeing Scale, *WSAS* Work and Social Adjustment Scale, *TES* Treatment Experience Scale, *HADS* Hospital Anxiety and Depression Scale

### Study quality

**Fig. 2:**
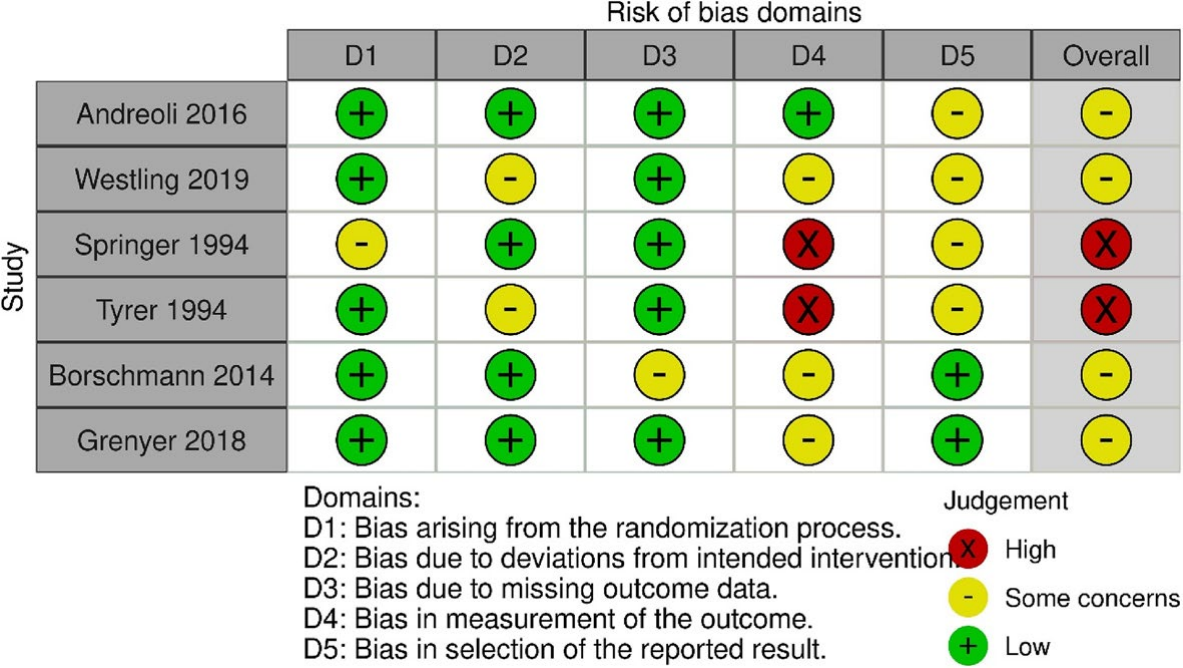
Assessment of the risk of bias in randomised controlled trials, using the Rob2 tool

**Fig. 3:**
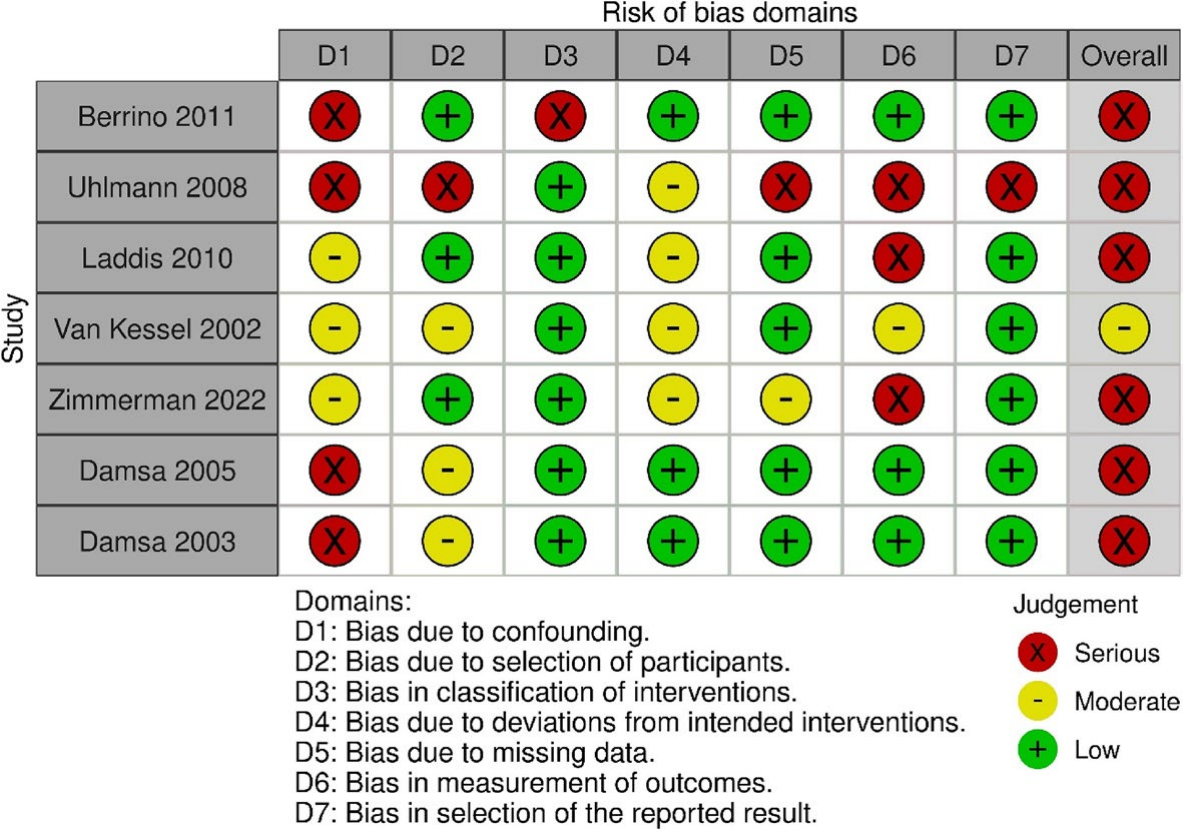
Assessment of risk of bias of non-randomised controlled studies using Robins-I tool

Of the six randomised controlled trials included, no studies were assessed as being at low risk of bias, four were assessed as having ‘some concerns’ regarding bias, and two were considered at high risk of bias (see Fig. 2), as the measurement of the outcome was thought to have potentially differed between groups. Six of the seven observational studies were assessed as being at serious risk of bias due to concerns across a range of domains (see Fig. 2). The remaining study considered at medium risk (see Fig. 3). The studies reporting changes in pre-post measures over time that were assessed using the Newcastle Ottawa Scale scored between 3 and 6, with these scores indicating either poor or fair quality (see Supplementary table, S1).

Assessment of quality of randomised controlled trials using RoB 2 tool:

Assessment of quality of observational controlled studies using the ROBINS-I tool:

The assessment of quality of observational studies reporting changes in pre-post outcomes over time was performed using Newcastle Ottawa Scale. See Supplementary tables, Table 4 for results.

### GRADE scoring: certainty of the evidence

For each model of care and group of outcomes, we reported our judgements of the certainty of the evidence based on the GRADE scoring system, below in Table 3.

**Table 3:** GRADE scoring: certainty of the evidence

Model	Outcomes assessed	Comparison group	Direction of effect found	Grade score
Hospital admission (no comparator models, all studies reporting changes over time)	Symptomatic improvement over time (range of measures including HoNOS)	No, change over time only	Improvement in symptoms scores	**Very low** ⊕ ◯◯◯
	Improvement in adaptive functioning over time	No, change over time only	Improvement in adaptive functioning	**Very low** ⊕ ◯◯◯
The brief admission model of hospital admission	Service use (days in hospital)	Treatment as usual	No effect of the intervention	**Moderate** ⊕ ⊕ ⊕ ◯
	Symptomatic improvement (range of measures)	No, change over time only	Improvement in symptoms scores	**Very low** ⊕ ◯◯◯
	NSSI and suicide attempts	Treatment as usual	No effect of the intervention	⊕ ⊕ ◯◯ **Low**
	Health Related QoL	No, change over time only	Improvement in QoL	**Very low** ⊕ ◯◯◯
	Therapeutic alliance	No, change over time only	Improvement in therapeutic alliance	**Very low** ⊕ ◯◯◯
Crisis teams	CGI	No, change over time only	Improvement in CGI scores	**Very low** ⊕ ◯◯◯
Acute day units	Symptomatic improvement (range of measures)	Telehealth version of ADU (1 study) Otherwise change over time only	Improvement in symptom scores	**Very low** ⊕ ◯◯◯
	Social participation	No, change over time only	Improvement in social participation	**Very low** ⊕ ◯◯◯
	Patient Satisfaction	Telehealth version of ADU	Higher satisfaction with in person versus telehealth ADU	**Very low** ⊕ ◯◯◯
Psychotherapies or psychosocial interventions: Outpatient based	Hospitalisation	TAU or TAU plus waitlist for psychological treatment	Reduction in % hospitalised, increased time to hospitalisation, reduction in number of admissions and bed days	**Moderate** ⊕ ⊕ ⊕ ◯
	Suicide attempt or suicide	TAU	Reduction in number of suicidal relapses and time to relapse	**Moderate** ⊕ ⊕ ⊕ ◯
	Symptomatic improvement	No, change over time only	Improvement in symptoms scores	**Very low** ⊕ ◯◯◯
Psychotherapies or psychosocial interventions: Based in emergency departments, general hospitals or psychiatric emergency services	Repeat suicide attempt	Historical TAU	Reduction in % of participants attempting suicide	**Very low** ⊕ ◯◯◯
	Hospitalisation	Historical TAU	Increased time to readmission and reduced days of hospitalisation	**Very low** ⊕ ◯◯◯
	Symptomatic improvement (range of measures)	No, change over time only	Improvement in symptoms scores	**Very low** ⊕ ◯◯◯
Psychotherapies or psychosocial interventions: groups delivered in inpatient services	Symptomatic improvement (range of measures)	‘Living well group’	No effect of the intervention	⊕ ⊕ ◯◯ **Low**
	Self-harm (including both suicide attempt and non-suicidal self-harm (NSSH)	No, change over time only	Reduction in frequency of self harm	**Very low** ⊕ ◯◯◯
Mother and Baby Units	GAF	No, change over time only	Reduction in score from discharge to 3 months	**Very low** ⊕ ◯◯◯
Joint crisis planning	Symptomatic improvement	Treatment as usual	No effect of the intervention	⊕ ⊕ ◯◯ **Low**
	NSSI		No effect of the intervention	⊕ ⊕ ◯◯ **Low**
Early Intervention Service	Symptomatic improvement	Treatment as usual (hospital-based services)	Greater improvement in the treatment as usual group	⊕ ⊕ ◯◯ **Low**
	Social functioning		Greater improvement in the treatment as usual group	⊕ ⊕ ◯◯ **Low**

*ADU*- acute day unit, *CGI* clinical global impression, *GAF* Global Assessment of Function, *GRADE* GRADE *Grading* of Recommendations, Assessment, Development, and Evaluations, *HoNOS* Health of the Nation Outcome Scales, *QoL* quality of life, *NSSH* Non-suicidal self-harm, *NSSI* Non-suicidal self-injury, TAU Treatment as Usual

See supplementary information 3 for detailed justification of judgements about the certainty of evidence for each model of care.

### Narrative synthesis of findings

A narrative synthesis was performed with study findings categorised by model of care and then subcategorised by outcome. Within models of care and outcome, contributing studies are separated into: a) studies comparing service models and b) studies reporting progress over time (pre-post outcomes for example from admission to discharge) without a comparison group.

### Acute psychiatric hospital admission

(See supplementary tables, Table S2).

Study types: we found seven studies that reported outcome data following hospital admission. No randomised trials were found for hospital admission and only one study included a comparison group, reporting outcomes before and after implementation of a specialised assessment ward. The remaining studies were cohort studies reporting changes in pre-post outcomes from admission to discharge.

There was some heterogeneity in the interventions offered, as reflected in the narrative synthesis. The majority of studies described general adult acute admissions. One studied a specialised ward for young adults (aged 18–24 years) [32], one ward was specifically aimed at treating mood disorders [59], one studied a specialised admission ward that had been introduced for people with a ‘personality disorder’ diagnosis [53] and the remainder studied general acute psychiatric wards.

Symptomatic outcomes:

- Studies comparing service models: A comparison of symptomatic improvement before and after implementing a specialised assessment ward for people with a ‘personality disorder’ diagnosis found no significant effect of implementing the intervention on improvement in participant’s brief symptom inventory scores compared to previous standard acute psychiatric admission [53]. This study did show an improvement in symptom scores over time in both groups.
- Studies reporting progress over time without a comparison group: Four studies reporting changes in pre-post outcomes reported that people with a diagnosis of a ‘personality disorder’ showed improvements on scores of symptom severity from admission to discharge [37, 38, 54, 59]. One study found that young people with a diagnosis of a ‘personality disorder’ showed a clinically significant improvement on the HoNOS score from admission to discharge on a specialised young adult ward, but authors commented that they showed a higher level of impairment on discharge than other diagnostic groups [32]. One study found that participants showed an improvement in adaptive functioning from pre-admission to six months after discharge [45].

Other outcomes:

- Studies comparing service models: ward atmosphere as rated by service users was found to improve after implementation of a specialised assessment ward for people with a ‘personality disorder’ diagnosis [53].

Summary:﻿﻿﻿ Included studies appeared to show that people who have been given a diagnosis of a ‘personality disorder’ can on average demonstrate improvement in measures of psychiatric symptom during hospital admission. Due to lack of controlled studies there was no information found about how outcomes following hospital admission compare to not being admitted to hospital or being supported in the community by alternatives forms of crisis care. The certainty of the evidence that acute psychiatric hospital admission reduces psychiatric symptoms or improves adaptive functioning for this population was judged to be very low, with no comparisons between service models or with no care possible.

### Brief admission

(see Supplementary tables, Table S3):

Study types: Six studies were found that reported outcomes following a brief admission intervention: 1 RCT [9], one non randomised study with a contemporaneous matched control group [55] and four cohort studies reporting changes in pre-post outcomes over time without comparison groups [29, 36, 41, 46]. The brief admission category included models of care referred to as ‘brief admission’ which has been previously described by Helleman [62], ‘patient initiated brief admission’ [36], ‘brief planned admissions’[55], ‘preventative psychiatric admission’[41, 46] and short psychiatric admissions (5–6 days) [29]. Brief admission models of care showed some variation but all investigated the impact of short (less than 14 days, most referring to admissions of 3–5 days) psychiatric admissions for people with a diagnosis of a ‘personality disorder’ as opposed to open ended general admissions. Additionally, the majority of studies referred to a model of care designed specifically for people with a ‘personality disorder’ diagnosis, where thought was given to developing a more positive therapeutic alliance than in general psychiatric admissions [9, 36, 46] a treatment contract was negotiated in advance [9, 36, 41, 46, 55], admission was through self-referral ([9, 36, 46]) and once admitted more autonomy was given to the participant than in a general psychiatric admission [36, 53]. The model in the Koekkek et al. study was different in that participants were admitted to hospital preventatively at prespecified intervals, aiming to recognise the person’s need for care and removing power struggles at times of crisis, whilst unlearning the association between crises and admission [41]. However, we included it in the brief admission category as it shared the following features with other models: admissions were short (for example 2–3 days), a treatment contract was negotiated in advance and it was a novel approach designed specifically for people with a ‘personality disorder’ diagnosis that aimed to develop a more positive therapeutic alliance than with general psychiatric admission.

Service use outcomes:

- Studies comparing service models: the only RCT identified found a significant reduction in days in hospital over time in both groups but no significant difference compared to treatment as usual (use of standard inpatient admissions) [9]. The non-randomised controlled study found a reduction in days in hospital in the brief admission group but not in the treatment as usual group (use of standard inpatient admissions), but no statistically significant between group difference [55]. One study of a preventative psychiatric admission program found no significant difference in mean days in hospital or outpatient contacts at 6 months before and after implementation of the intervention, compared to use of standard inpatient admissions [41].

Other outcomes:

- Studies comparing service models: The RCT found a reduction in non-suicidal self injury events over time in the intervention and treatment as usual groups, but no statistically significant between group difference [9]. A significant improvement in therapeutic alliance, as rated by the therapist, was found after the introduction of preventative psychiatric admissions in one study compared to a historical control group [41].
- Studies reporting progress over time without a control group: Two cohort studies reporting pre-post outcomes found a reduction in symptom scores from admission to discharge [29].

Summary: The included studies found reductions in service use and non-suicidal self-injury over time in participants who received the brief admission model, but the randomised study and non-randomised controlled study found no effect of brief admission compared to treatment as usual. The controlled studies did not report on any symptomatic measures, service satisfaction or any other outcomes that would reflect the service users’ experiences of the care and services. The certainty of the evidence that brief admission does not reduce service use in this population compared to treatment as usual was judged to be moderate. The certainty of the evidence that brief admission reduces NSSI and suicide attempts compared to treatment as usual was judged to be moderate. The certainty of the evidence that brief admission reduces symptoms, improves health related quality of life and therapeutic alliance was judged to be very low, with no comparisons with other models of care or no care possible.

### Crisis teams

(see Supplementary tables, Table S4).

Study types: We found only one paper meeting the inclusion criteria that reported outcomes following use of crisis teams. This was a cohort study reporting outcomes at the start and end of intensive home treatment [51]. The authors note that 18% of patients contributed 51% of referrals to the intensive home treatment team.

Symptomatic improvement:

- Studies comparing service models: no studies found
- Studies reporting progress over time without a comparison group: 60% of participants showed improvement on the CGI measure from admission to discharge [64].

Summary: The literature investigating crisis teams is very sparse with limited findings. The certainty of the evidence for the effect of crisis teams on clinical global impression scores was judged to be very low with no comparisons between models possible.

### Acute day units/day hospitals/acute partial hospitalisation

(see Supplementary tables, Table S5),

Study types: Six studies reported outcomes following use of acute day units, including one non-randomised controlled study that compared outcomes before and after implementation of a new service and five cohort studies reporting change in pre-post outcomes over time, without a comparison group.

The interventions delivered within the setting of the acute day units showed the following common features: participants attended a psychologically informed program of activities and therapy sessions including both group sessions and individual support, participants attended the day unit during working hours and returned home at night, acute day units accepted referrals following emergency psychiatric presentations and the interventions were supported by a multidisciplinary team. Interventions varied in length from five days to eight weeks. Psychological models used in delivering the therapeutic component included DBT [58], Cognitive Behavioural Therapy (CBT) [56], Acceptance and Commitment Therapy (ACT) [60] and mindfulness [60].

Symptomatic improvement:

- Studies comparing service models: One non-randomised controlled study compared symptomatic improvement between groups treated before and after conversion of an in-person day hospital program to a telehealth program during the Covid-19 pandemic [60]. The authors found that both groups showed similar improvements on symptom scores for depression; there was a difference between the two groups only on the subscale of functioning (greater improvement in virtual group) and anger (greater improvement in in person group).
- Studies reporting progress over time without a comparison group: Four studies reported improvements in scores on symptomatic measures from admission to discharge [43, 48, 56, 60]. Two studies reported changes in symptomatic measures from discharge to 3 months follow up, one of which found a significant improvement in scores, although noted that scores on many measures remained within the moderate to severe clinical range on discharge [58], and one study found a non-significant deterioration in scores from discharge to three month follow up [43]. Other outcomes: One study found an improvement in social participation [43] from admission to discharge.

Summary: The lack of controlled studies means that conclusions cannot be drawn about the effectiveness of acute day units for people with a diagnosis of a ‘personality disorder’ compared with other types of care or no care. Telehealth versions of acute day units may show similar results to in person versions. Included participants appeared to show improvements in symptom scores and measures of social participation after using acute day hospitals but this was not compared to other models of care. The certainty of evidence for the effect of acute day units on symptom score, social participation and patient satisfaction in this population was judged to be very low, with no comparisons between models possible.

### Outpatient-based crisis-focused psychotherapies or psychosocial interventions

(see Supplementary tables, Table S6).

Study types: Five studies were found including one RCT, one cluster RCT and three cohort studies reporting pre-post outcomes over time without comparison groups. All studies reported findings for models of psychological or psychosocial intervention that could be initiated urgently in crisis and were delivered in an outpatient settings, but did not provide other features of day hospital programs such as multidisciplinary teams and occupational therapy. Some treatment programs accepted referrals following an initial crisis inpatient admission of 1–2 days [28, 44], but following this treatment was on an outpatient basis. Length of intervention ranged from one month to three months. One study described a program based on DBT [44], one focused on relationship losses [28], two on crisis management [39, 40] and one on crisis psychotherapy [47]. Narrative synthesis findings below are described with reference to the type of service provided.

Hospitalisation:

- Studies comparing service models: One RCT found lower rates of repeat hospitalisation over 3 months in those undergoing ‘abandonment psychotherapy’; a 3 month twice weekly manualised psychotherapy focusing on relationship losses delivered by either a nurse or therapist in conjunction with medication and a risk management plan [28] compared to treatment as usual (intensive community treatment). This intervention was delivered to participants with comorbid major depressive disorder and ‘borderline personality disorder’ after presenting to the emergency department following a suicide attempt or act of self-harm severe enough to require emergency medical or surgical treatment. In this model there was the option that participants could be initially admitted for a brief psychiatric hospitalisation of around four days if they met severity criteria, following which they could urgently commence the outpatient intervention. One cluster RCT of the introduction of a crisis focused brief psychological intervention, involving one month of weekly crisis focused contact, found a significantly greater reduction in mean days in hospital in the intervention site compared to the treatment as usual site once the active phase of the trial was initiated [39], but no difference in total number of admissions.

Suicide attempt:

- Studies comparing service models: The RCT found lower rates of repeat suicide attempts in the group receiving the intervention compared to the treatment as usual group and a greater survival time to ‘suicidal relapse’, which was defined as any episode of suicidal ideation, with or without self-harm that was severe enough to require additional intensive psychiatric care [28].

Symptomatic measures:

- Studies comparing service models: no studies found
- Studies reporting progress over time, without a comparison group: Three cohort studies found a significant reduction in scores on symptomatic measures from the start until the end of treatment. These studies evaluated a 3 week intensive outpatient DBT program [44], a 10 week crisis psychotherapy program [47] and the same brief psychological intervention studied in the cluster RCT [40].

Summary: Both randomised studies showed a promising effect of outpatient-based crisis focused psychotherapies initiated at the time of a crisis. The trial of ‘abandonment psychotherapy’ was of overall higher quality and reported a wider range of outcomes, but is a model that does not appear to be in wide use outside the study site and so replication studies are not available. The certainty of the evidence that the included outpatient psychological or psychosocial treatments reduce hospitalisation and repeat suicide attempts for this population, compared to treatment as usual, was judged to be moderate. The certainty of the evidence that the included models improve symptom scores was judged to be very low, with no comparisons with other models of care possible.

### Psychological or psychosocial therapies based in Emergency Departments or Psychiatric Emergency Services

(see Supplementary tables, Table S7).

Study types: Five studies were found studying the introduction of a crisis intervention service as a follow up service from the Emergency Department [30], a crisis stabilisation unit within a psychiatric emergency service (PES) [33], a model of crisis intervention developed in French speaking countries [35, 61] and crisis intervention based on the ‘Cape Cod Model’ within a crisis stabilisation unit. All services focused on a psychological intervention delivered over a few days whilst participants stayed in a crisis stabilisation unit or short stay crisis bed, situated within a psychiatric emergency service or general hospital. Treatments focused on crisis and problem solving [33], emotional dysregulation and internal and interpersonal conflicts that triggered the crisis [35, 65]. Study designs included one non-randomised controlled study with contemporaneous controls, three non-randomised studies with historical controls, one cohort study reporting changes in pre-post outcomes over time.

Symptomatic measures:

- Studies comparing service models: The non-randomised controlled study with contemporaneous controls found greater improvements in symptomatic measures in the intervention group than control group [42].

Suicide attempts and hospitalisation:

- Studies comparing service models: One non-randomised controlled study with a historical control group found significantly fewer repeat suicide attempts at 3 months, significantly longer time to readmission and fewer days of hospitalisation at 3 months after the introduction of a crisis intervention in an emergency department [30]. The other two non-randomised controlled studies with historical controls found reduced rates of hospitalisation after introduction of a crisis intervention model in the emergency department [34, 35, 66].

Summary: Studies found promising improvements in outcomes following introduction of crisis focused psychosocial therapies in the emergency department for people with a diagnosis of a ‘personality disorder’. A lack of randomised studies means that firm conclusions cannot be drawn about the effectiveness of these interventions. The certainty of the evidence for psychological or psychosocial therapies based in emergency departments or psychiatric emergency services reducing suicide attempts, hospitalisation and improving symptom scores for this population was judged to be very low, with no comparisons between models of care possible.

### Psychotherapy groups on inpatient wards

(see Supplementary tables, Table S8).

Study types: Two studies, including one RCT and one cohort study with pre-post outcomes over time, both studied a group intervention based on DBT skills on inpatient wards. DBT-based groups were delivered in two week cycles of repeating sessions, with one study offering up to 6 weeks of sessions [31].

Symptomatic measures:

- Studies comparing service models: a RCT showed no significant difference in symptomatic outcomes in participants undergoing a 10 session DBT skills based group compared to a ‘living well’ group, except on ‘locus of control’ subscale [50]. This RCT was conducted in 1996 and so there may have been substantial developments to the DBT group model since it was performed.
- Studies reporting progress over time, without comparison groups: one study showed significant improvement in symptomatic measures [31] from start until the end of the group program.

Other outcomes: one study found a reduction in the frequency of self-harm from baseline, post intervention and at 3 months, but did not compare findings to a comparison group.

Summary: The certainty of the evidence was judged to be low for DBT based short term psychotherapy groups delivered on inpatient wards resulting in improvement on symptoms scores, compared to a ‘living well group’. The certainty of evidence of DBT based groups reducing self-harm (both suicide attempts and NSSH) was judged to be very low and no comparison to other models of care or no care was possible.

### Mother and Baby Units

(see Supplementary tables, Table S9).

Summary: we found only one study that reported change in symptoms scores over time without a comparison group. The study reported a deterioration on the Global Assessment of Functioning and CARE index (a measure of mother infant relationship) from discharge to 3 months post discharge [57]. The study did not measure change from admission to discharge. The certainty of the evidence for people with a ‘personality disorder’ diagnosis discharged from Mother and Baby Units showing a reduction in functioning from discharge to follow up was judged to be very low.

### Joint crisis plans

(see Supplementary tables, Table S10).

Summary: One pilot RCT of joint crisis plans found no significant differences in self-harm outcomes or in symptomatic measures between the intervention and treatment as usual groups [11], but did conclude that the intervention was feasible. This was a pilot RCT and was powered to detect a threefold difference in proportion of participants who self-harmed between groups, therefore the study may have been underpowered to detect a difference in symptom scores or smaller differences in the rate of self-harm. The certainty of evidence was therefore graded as low.

### Community Early Intervention Service

(See supplementary tables, Table S11).

Summary: One RCT compared a community early intervention service (predating the development of the current psychosis-focused early intervention model) to hospital-based services. The original RCT included a transdiagnostic group of participants but the study focused on a smaller subgroup (*n* = 50) with a ‘personality disorder’ diagnosis. The intervention involved 12 weeks of multidisciplinary community-based care following presentation as a ‘psychiatric emergency’ to a general hospital. The authors state that those without a ‘personality disorder’ diagnosis improved to a much greater extent on symptom scores in the community service than in the hospital service, with opposite effects shown for those with a ‘personality disorder’ diagnosis, although the data presented suggests that the symptom scores of people with a ‘personality disorder’ improved to a similar degree in both services. Social function scores for those with a ‘personality disorder’ diagnosis remained stable over time in the EIS group but improved over time in the standard hospital-based service group [52]. The certainty of evidence that participants with a ‘personality disorder’ diagnosis experience greater improvements in symptomatic measures and social function in hospital-based services compared to an early intervention service model was judged to be low.

## Discussion

The number of eligible studies identified in this review was low considering the wide inclusion criteria, demonstrating the lack of evidence in this field. Overall, the quality of evidence available was low, with only six randomised studies found across all models and a large number of uncontrolled studies. The majority of studies were judged to be at high risk of bias and the certainty of evidence was judged to be low or very low for all models except for brief admission and outpatient-based crisis-focused psychosocial and psychological crisis interventions. The literature for acute care is smaller than for community care for people with this diagnosis, which is also under researched [67]. The review included studies that were found in the previous more focused reviews [10, 12], but also included a wider range of literature.

There were a large number of studies found reporting only changes in pre-post measures over time without a comparison group, particularly for the outcome of symptomatic improvement. The majority of these studies reported improvements in symptom scores after using crisis care, whether that was hospital admission, crisis teams, acute day units or psychotherapies. Result of these uncontrolled studies have very limited implications however, as it would be expected that participant will show an improvement over time, particularly when baseline measures are performed during an acute crisis. The lack of comparisons between different crisis models means that there is little information about the degree of improvement when using one service over another or compared to not accessing crisis services.

The literature on crisis teams is particularly sparse and low quality, with only one study identified, and this reported only changes in pre-post outcomes over time. This is an important evidence gap given that crisis teams are recommended in the most recent UK NICE guidelines as a first line intervention in a crisis for people with a ‘borderline personality disorder’ diagnosis who may need hospitalisation [16]. A previous systematic review of qualitative literature also found no published qualitative studies exploring experiences of people with a ‘personality disorder’ diagnosis using crisis teams [68].

Two outpatient-based crisis-focused psychotherapy models were the only models of care identified that were supported by RCT evidence of a statistically and clinically significant benefit compared to treatment as usual. These were a 4-session crisis focused psychosocial intervention from Australia [39] and a 3-month intensive manualised therapy called ‘abandonment psychotherapy’ [28], designed to be used following presentation in crisis and focusing on relationship losses. Both interventions were delivered within a system of acute care that provided concurrent risk assessment and the option of brief hospitalisation, medication and more intensive input if the individual needed this. The Australian intervention was also offered within a stepped care model that offered referral to longer term treatments. Crisis-focused psychological and psychosocial outpatient interventions that can be delivered as part of an acute care system may therefore be a promising area for future research. That these services do not operate as standalone interventions but require interaction with the rest of an acute care system may limit generalisability to contexts where acute care options are not extensively developed.

The evidence we identified that investigates acute hospitalisation for people with a ‘personality disorder’ diagnosis in crisis was of low quality for all outcomes. The lack of certainty in the evidence is important, as many clinicians and policy makers believe that hospitalisation for people with a ‘personality disorder’ diagnosis can cause iatrogenic harm and can cause a deterioration in symptoms and in risk [19, 69], although much of the academic literature is more equivocal on this question [11, 16, 70, 71]. Evidence that demonstrates clearly whether and when acute psychiatric admissions might be helpful or harmful was not found in the studies identified by this review. Four included studies reported an improvement in symptom scores during hospital admission [37, 38, 53, 54] and no studies reported a deterioration, although most studies did not capture adverse events or other possible negative outcomes such as self-harm. Improvements in symptoms between admission and discharge tended to be reported in studies, but whether hospitalisation had contributed positively to theseimprovements was unclear, as improvements over time may well have occurred in other settings and people are more likely to be discharged at a point when improvement has been observed. Negative experiences that may result from hospitalisation, such as violence, coercion and other traumatising experiences [72], tended not to be reported in the studies we retrieved. Based on the quality of evidence available, conclusions cannot be drawn on the effectiveness of acute hospital admission in crisis for people with a ‘personality disorder’ diagnosis. The current lack of evidence supports neither offering acute hospital admissions nor refraining from doing so wherever possible. The basis of beliefs that hospitalisation is harmful is questionable given the limitations of the evidence regarding harms. We would suggest that clinical recommendations should reflect this uncertainty and care should be taken to reduce the impact of stigma against this group in influencing treatment choices. Given that people with this diagnosis continue to be offered acute admissions, there is a need to develop and implement co-produced strategies to improve experiences and outcomes of inpatient admissions when they occur. This review did not compare outcomes for people with a ‘personality disorder’ diagnosis to other diagnostic groups using acute care, which could be useful further work.

The evidence for brief admission was considered separately from general acute psychiatric hospital admission, as brief admission in the majority of studies referred to a distinct model tailored to people with a diagnosis of a ‘personality disorder’ in which individuals at high risk of admission to hospital entered into a pre-agreed treatment contract and modifications were made to the inpatient experience, for example the person managed their own medications. Although the quality of evidence for brief admission was of higher quality than acute psychiatric hospital admission, it is not possible to draw generalisable conclusions about hospitalisation from the randomised controlled trial of brief admission, as both the intervention and treatment as usual groups also experienced general psychiatric hospital admissions during the follow-up period.

It was not possible to perform a meta-analysis in this review due to the wide range of outcome measures used in different studies. This was particularly the case for measures used to quantify symptomatic improvement, where there were very few studies with any overlap in the measures used. Studies tended to use a large battery of different measures, covering domains such as anxiety, depression, self-harm, hopelessness, and social participation. The suitability of measures that were developed to focus on relapse and recovery in depression and anxiety when applied to people with a ‘personality disorder’ diagnosis is not known. There is debate about the suitability of traditional concepts of recovery for people with a diagnosis of a ‘personality disorder’, for whom the course of a person’s difficulties might not be characterised by episodic relapses and linear, sustained improvements in symptoms, but living with more longstanding difficulties and more frequent fluctuations that may require crisis support, but with gradual improvements in the development of relationships, social functioning and sense of self alongside this [73–75]. The need for a wide battery of measures to capture different aspects of symptoms and social functioning may place a larger burden on participants, potentially reducing recruitment and retention in studies, particularly of those who are most unwell. In contrast services have previously been found to focus too narrowly on improvements in self-harm and emotional dysregulation [75]. The development of a core set of outcome measures should be considered for future studies that are meaningful for people with a ‘personality disorder’ diagnosis and can reflect the fluctuations in symptom severity that can occur. The most recent UK national guidelines (NICE) for the care of people with a ‘borderline personality disorder’ diagnosis recommend the development of an agreed set of outcome measures as a priority for future research [16]. The International Consortium for Health Outcomes Measurement (ICHOM) conducted a Delphi Study published in 2021 aimed to develop a standard set of patient reported outcomes for ‘personality disorder’ but only one measure recommended in this paper was found in the literature for this review (WHODAS) and it is unclear the extent to which this has been adopted in ongoing research [76].

Other than measures of symptomatic severity, the next most commonly reported groups of outcomes were service use, including hospitalisation and days in hospital. There was a lack of information across studies about experiences of care of both staff and people using services. The subjective experience of care seems particularly important in a crisis where the aim of services is to provide support during a period of distress. A meta-synthesis we performed of qualitative studies describing experiences of crisis care for this group found a paucity of literature, but with participants emphasising the importance of the perceived attitudes of staff, skills in communication and the quality of relationships formed between crisis service staff and service users as being central to the experience of crisis services, regardless on the model of care used [15]. The lived experience working group for this review expressed scepticism for the expectation that a person should improve on quantitative symptomatic measures over a short space of time in a crisis and instead said they hoped crisis services could offer the feeling of being held and known about by a supportive team that can offer contact and containment whilst they experienced a crisis and began recovery. Efforts should be made in future research to capture outcomes measures more suitable for this group of people.

### Strengths and limitations

The review has a wide inclusion criteria and reports findings for a wide range of models. The studies were heterogeneous in design and in the outcomes reported and so it was not possible to perform a meta-analysis. This limits the conclusions that can be drawn about the effectiveness of the available models of crisis care for people with complex emotional needs. However, it does describe and synthesise the literature that is available.

The review did not include studies that investigated services that offer an initial assessment in crisis but no ongoing crisis care, such as emergency departments, psychiatric liaison teams (unless brief admission or some follow up care was offered) or crisis lines as these were not felt to be comparable to other crisis and acute care models for the purpose of this review. However for some in some circumstances attending emergency departments and engaging with a psychosocial assessment may be a crisis intervention in its own right [77]. Additionally, the review was not able to include models for which there was no studies found, but which might be promising in the care of people with a diagnosis of a ‘personality disorder’, such as crisis houses and crisis cafes. This review did not compare outcomes following use of acute care for people with a ‘personality disorder’ diagnosis in comparison to other diagnostic groups such as depression: a review of such evidence would be of interest to inform discussions about whether people with a ‘personality disorder’ diagnosis experience specific harmful or reduced positive—effects from admission.

The inclusion criteria only required > 50% of participants to have been given a diagnosis of a ‘personality disorder’, therefore there are participants included in these studies with other diagnoses. Due to the service settings, the majority of studies that included participants with other diagnoses, participants had diagnoses of mood, anxiety disorders or difficulties such as complex PTSD. This review has synthesised studies that included participants with a mixture of ‘borderline personality disorder’ diagnosis and other subtypes of the diagnosis as one group. We felt this was justified given that many people meet the criteria for multiple subtypes and this has been recognised in changes to the ICD-11 which will no longer specify subtypes in the diagnosis [78]. All studies did capture people presenting with difficulties associated with ‘personality disorder’ diagnosis in crisis, but many included studies did not describe use of a validated instrument to make the diagnosis of a ‘personality disorder’. There was therefore also no information available about how severity of a person’s difficulties might affect the effectiveness and choice of crisis care options.

The language of studies was not limited to English, but search terms were only defined in English.

## Conclusions

The literature in this field is sparse and of overall low quality. There were no high-quality studies that investigated outcomes following crisis teams or hospital admissions for this group despite these being the core models of care available in most UK and European mental health services. There were few comparisons available that provide any information about which types of models of care might be more or less effective for this group in crisis. Studies of crisis focused psychosocial and psychological interventions were small in number but showed some promising results. Future work should aim to provide better information on which to base clinical decisions for this group in a crisis, through higher quality studies and considering the use of core outcomes measures that are meaningful for people with a ‘personality disorder’ diagnosis that would allow comparison of different models.

## Lived experience commentary (Eva Broeckelmann)

"From a lived experience perspective, the lack of evidence *against* hospitalisation for people with a ‘personality disorder’ diagnosis in crisis might arguably be the most important finding of this review. It appears that the common belief amongst mental health professionals that admissions are counterproductive for this entire group of service users is in fact so far nothing but an unsubstantiated myth.

However, while it is certainly validating to know that the evidence base does not support exclusion on the basis of this stigmatising label, that doesn’t change the harsh reality that too many people with CEN continue to suffer the consequences of such discriminatory practices, which are not only limited to hospitalisation, but extend into other forms of crisis care as well.

As the studies included in this review confirm that symptomatic improvements can in fact be achieved with a variety of interventions, there really is no justification for a blanket exclusion of service users with CEN from desperately needed support in crisis.

The lack of direct comparisons between different models of crisis care seems significantly less important, especially considering the highly subjective nature of a ‘personality disorder’ diagnosis and large heterogeneity amongst service users with CEN. As there simply is no ‘one-size-fits-all’, crisis care – like any other mental health service – should always be flexible and person-centred, providing options tailored to meet individual needs and empower service users instead of relying on professionals to make decisions about us, without us.

Maybe it is no coincidence that the interventions that showed the most significant benefit were outpatient-based crisis-focused psychotherapy models that operated within integrated systems which were adaptable and provided opportunities for more collaborative care.

Ultimately, instead of more RCTs with outcome measures of questionable suitability for people with CEN, future research in this area should rather focus on qualitative studies of service user experiences of crisis care as well as addressing the underlying stigma and pejorative attitudes that continue to result in malignant alienation of service users on the basis of a ‘personality disorder’ diagnosis.

After all, when someone seeks help in a crisis, any offer of support is better than none."

### Supplementary Information

**Additional file 1.** Search terms used, by database.

**Additional file 2: Table S1.** Assessment of the quality of studies reporting pre and post intervention outcomes over time, without a comparison group, using the Newcastle Ottowa Scale. **Table S2.** Outcomes: hospital admission. **Table S3.** Outcomes: brief admission. **Table S4.** Outcomes: Crisis teams. **Table S5.** Outcomes: Acute Day Unit. **Table S6.** Outcomes: Crisis-focused psychotherapies or psychosocial interventions. **Table S7.** Outcomes: Crisis-focused psychotherapies or psychosocial interventions In emergency departments or psychiatric emergency services. **Table S8.** Outcomes: Crisis-focused psychotherapies or psychosocial interventions. **Table S9.** Outcomes: Mother and Baby Units. **Table S10.** Outcomes: Joint crisis plans. **Table S11.** Outcomes: Early Intervention Service.

**Additional file 3:** **Supplementary file 3.** GRADE scoring criteria for interventional studies evaluating crisis and acute mental health care for people with complex emotional needs who may have a diagnosis of a ‘personality disorder’.

**Additional file 4:** **Supplementary File 4 (S4).** Illustrative examples of quality assessment tools (Rob2 and Robins-I).

## Data Availability

All data generated or analysed during this study are included in this published article and its supplementary information files.
