# Supplementary material for: Crisis and acute mental health care for people who have been given a diagnosis of a ‘personality disorder’: a systematic review
Source: BMC Psychiatry. 2023 Oct 5;23:720. doi: 10.1186/s12888-023-05119-7 (PMC10552436; doi:10.1186/s12888-023-05119-7)
Supplement: Supplementary file 1 — Additional file 1. Search terms used, by database. [file 12888_2023_5119_MOESM1_ESM.docx]

**Supplementary file 1: Search terms used, by database**

**Database 1:** Ovid MEDLINE(R) ALL

Date parameters: articles published between 1946 and the 1^st^ March 2022

Date of search: 2^nd^ March 2022

| **#** | **Query** |
| --- | --- |
| 1 | exp Personality Disorders/ or personality disorder*.mp. |
| 2 | "complex emotional need*".mp. |
| 3 | ("complex needs" and ("mental health" or "emotion*")).mp. [mp=title, abstract, original title, name of substance word, subject heading word, floating sub-heading word, keyword heading word, organism supplementary concept word, protocol supplementary concept word, rare disease supplementary concept word, unique identifier, synonyms] |
| 4 | emotion* dysreg*.mp. [mp=title, abstract, original title, name of substance word, subject heading word, floating sub-heading word, keyword heading word, organism supplementary concept word, protocol supplementary concept word, rare disease supplementary concept word, unique identifier, synonyms] |
| 5 | personality difficult*.mp. [mp=title, abstract, original title, name of substance word, subject heading word, floating sub-heading word, keyword heading word, organism supplementary concept word, protocol supplementary concept word, rare disease supplementary concept word, unique identifier, synonyms] |
| 6 | personality dysfunction*.mp. [mp=title, abstract, original title, name of substance word, subject heading word, floating sub-heading word, keyword heading word, organism supplementary concept word, protocol supplementary concept word, rare disease supplementary concept word, unique identifier, synonyms] |
| 7 | affect regulation disorder.mp. [mp=title, abstract, original title, name of substance word, subject heading word, floating sub-heading word, keyword heading word, organism supplementary concept word, protocol supplementary concept word, rare disease supplementary concept word, unique identifier, synonyms] |
| 8 | emotion* instab*.mp. [mp=title, abstract, original title, name of substance word, subject heading word, floating sub-heading word, keyword heading word, organism supplementary concept word, protocol supplementary concept word, rare disease supplementary concept word, unique identifier, synonyms] |
| 9 | borderline state*.mp. [mp=title, abstract, original title, name of substance word, subject heading word, floating sub-heading word, keyword heading word, organism supplementary concept word, protocol supplementary concept word, rare disease supplementary concept word, unique identifier, synonyms] |
| 10 | Cluster A personalit*.mp. [mp=title, abstract, original title, name of substance word, subject heading word, floating sub-heading word, keyword heading word, organism supplementary concept word, protocol supplementary concept word, rare disease supplementary concept word, unique identifier, synonyms] |
| 11 | Cluster B personalit*.mp. [mp=title, abstract, original title, name of substance word, subject heading word, floating sub-heading word, keyword heading word, organism supplementary concept word, protocol supplementary concept word, rare disease supplementary concept word, unique identifier, synonyms] |
| 12 | (borderline personalit* or emotion* unstable personalit* disorder or impulsive personalit* or histrionic personalit* or narcissistic personalit* or antisocial personalit* or dissocial personalit* or paranoid personalit* or schizoid personalit* or schizotypal personalit* or avoidant personalit* or anxious personalit* or dependent personalit* or obsessive compulsive personalit* or anankastic personalit* or sociopathic personalit* or mixed personality disorder*).mp. [mp=title, abstract, original title, name of substance word, subject heading word, floating sub-heading word, keyword heading word, organism supplementary concept word, protocol supplementary concept word, rare disease supplementary concept word, unique identifier, synonyms] |
| 13 | Crisis Intervention/ or crisis intervention*.mp. |
| 14 | exp Emergency Services, Psychiatric/ or emergency psychiatric service*.mp. |
| 15 | brief intervention*.mp. |
| 16 | ("crisis team*" or "home treatment team*").mp. [mp=title, abstract, original title, name of substance word, subject heading word, floating sub-heading word, keyword heading word, organism supplementary concept word, protocol supplementary concept word, rare disease supplementary concept word, unique identifier, synonyms] |
| 17 | (Crisis house or recovery house or sanctuar* or "safe haven" or "alternative* to hosp*" or "alternative* to admission").mp. [mp=title, abstract, original title, name of substance word, subject heading word, floating sub-heading word, keyword heading word, organism supplementary concept word, protocol supplementary concept word, rare disease supplementary concept word, unique identifier, synonyms] |
| 18 | (crisis cafe* or crisis pad*).mp. [mp=title, abstract, original title, name of substance word, subject heading word, floating sub-heading word, keyword heading word, organism supplementary concept word, protocol supplementary concept word, rare disease supplementary concept word, unique identifier, synonyms] |
| 19 | ((hospital or admission or admitt* or ward) and (mental health or psychiatr*)).mp. [mp=title, abstract, original title, name of substance word, subject heading word, floating sub-heading word, keyword heading word, organism supplementary concept word, protocol supplementary concept word, rare disease supplementary concept word, unique identifier, synonyms] |
| 20 | exp Hospitals, Psychiatric/ |
| 21 | ("acute day unit*" or "acute day hospital*" or "acute day treatment*").mp. [mp=title, abstract, original title, name of substance word, subject heading word, floating sub-heading word, keyword heading word, organism supplementary concept word, protocol supplementary concept word, rare disease supplementary concept word, unique identifier, synonyms] |
| 22 | ("crisis service*" or "crisis care").mp. [mp=title, abstract, original title, name of substance word, subject heading word, floating sub-heading word, keyword heading word, organism supplementary concept word, protocol supplementary concept word, rare disease supplementary concept word, unique identifier, synonyms] |
| 23 | 1 or 2 or 3 or 4 or 5 or 6 or 7 or 8 or 9 or 10 or 11 or 12 |
| 24 | 13 or 14 or 15 or 16 or 17 or 18 or 19 or 20 or 21 or 22 |

**Database 2:** APA PsycInfo

Date parameters: 1806 to February week 08 2022

Date of search: 2^nd^ March 2022

1 personality disorder.mp. or exp Personality Disorders/

2 "complex emotional need*".mp.

3 ("complex needs" and ("mental health" or "emotion")).mp. [mp=title, abstract, heading word, table of contents, key concepts, original title, tests & measures, mesh word]

4 "emotion* dysreg*".mp. [mp=title, abstract, heading word, table of contents, key concepts, original title, tests & measures, mesh word]

5 "personality difficult*".mp. [mp=title, abstract, heading word, table of contents, key concepts, original title, tests & measures, mesh word]

6 "personality dysfunction*".mp. [mp=title, abstract, heading word, table of contents, key concepts, original title, tests & measures, mesh word]

7 "affect regulation disorder*".mp. [mp=title, abstract, heading word, table of contents, key concepts, original title, tests & measures, mesh word]

8 "emotion* instab*".mp. [mp=title, abstract, heading word, table of contents, key concepts, original title, tests & measures, mesh word]

9 borderline state*.mp. [mp=title, abstract, heading word, table of contents, key concepts, original title, tests & measures, mesh word]

10 borderline state.mp. or exp Borderline States/

11 "Cluster A personalit*".mp. [mp=title, abstract, heading word, table of contents, key concepts, original title, tests & measures, mesh word]

12 "Cluster B personalit*".mp. [mp=title, abstract, heading word, table of contents, key concepts, original title, tests & measures, mesh word]

13 (borderline personalit* or emotion* unstable personalit* disorder or impulsive personalit* or histrionic personalit* or narcissistic personalit* or antisocial personalit* or dissocial personalit* or paranoid personalit* or schizoid personalit* or schizotypal personalit* or avoidant personalit* or anxious personalit* or dependent personalit* or obsessive compulsive personalit* or anankastic personalit* or sociopathic personalit* or mixed personality disorder*).mp. [mp=title, abstract, heading word, table of contents, key concepts, original title, tests & measures, mesh word]

14 crisis intervention.mp. or exp Crisis Intervention/

15 ("crisis service*" or "crisis intervention service*" or "crisis care").mp. [mp=title, abstract, heading word, table of contents, key concepts, original title, tests & measures, mesh word]

16 exp Crisis Intervention Services/

17 exp Psychiatric Hospitals/

18 exp Psychiatric Units/

19 exp Psychiatric Hospital Admission/ or exp Psychiatric Hospitalization/

20 exp Brief Psychotherapy/ or brief intervention.mp.

21 ("crisis team*" or "home treatment team*").mp. [mp=title, abstract, heading word, table of contents, key concepts, original title, tests & measures, mesh word]

22 (Crisis house or recovery house or sanctuar* or "safe haven" or "alternative* to hosp*" or "alternative* to admission").mp. [mp=title, abstract, heading word, table of contents, key concepts, original title, tests & measures, mesh word]

23 (crisis cafe* or crisis pad*).mp. [mp=title, abstract, heading word, table of contents, key concepts, original title, tests & measures, mesh word]

24 ((hospital or admission or admitt* or ward) and (mental health or psychiatr*)).mp. [mp=title, abstract, heading word, table of contents, key concepts, original title, tests & measures, mesh word]

25 ("acute day unit*" or "acute day hospital*" or "acute day treatment*").mp. [mp=title, abstract, heading word, table of contents, key concepts, original title, tests & measures, mesh word] 34

26 1 or 2 or 3 or 4 or 5 or 6 or 7 or 8 or 9 or 10 or 11 or 12 or 13

27 14 or 15 or 16 or 17 or 18 or 19 or 20 or 21 or 22 or 23 or 24 or 25

28 26 and 27

**------------------**

**Database 3:** Cochrane CENTRAL

Date parameters: until March 2022

Date of search: 2^nd^ March 2022

ID Search Hits

#1 ("personality disorder"):ti,ab,kw (Word variations have been searched) in Cochrane Protocols, Trials 2817

#2 "complex emotional need*" 0

#3 "complex needs" and ("mental health" or "emotion") in Cochrane Protocols, Trials 33

#4 "emotion* dysreg*" in Cochrane Protocols, Trials 0

#5 "personality difficult*" in Cochrane Protocols, Trials 0

#6 "personality dysfunction*" in Cochrane Protocols, Trials 12

#7 emotion* instab* in Cochrane Protocols, Trials 198

#8 "borderline state*" in Cochrane Protocols, Trials 369

#9 "Cluster A personalit*" in Cochrane Protocols, Trials 0

#10 "Cluster B personalit*" in Cochrane Protocols, Trials 0

#11 "borderline personalit*" or "emotion* unstable personalit* disorder" or "impulsive personalit*" or "histrionic personalit*" or "narcissistic personalit*" or "antisocial personalit*" or "dissocial personalit*" or "paranoid personalit*" or "schizoid personalit*" or "schizotypal personalit*" or "avoidant personalit*" or "anxious personalit*" or "dependent personalit*" or "obsessive compulsive personalit*" or "anankastic personalit*" or "sociopathic personalit*" or "mixed personality disorder*" in Cochrane Protocols, Trials 0

#12 "crisis intervention" OR "crisis service*" OR "crisis care" in Cochrane Protocols, Trials 431

#13 "brief intervention*" in Cochrane Protocols, Trials 2217

#14 "crisis team*" or "home treatment team*" in Cochrane Protocols, Trials 26

#15 "Crisis house" or "recovery house" or sanctuar* or "safe haven" or "alternative* to hosp*" or "alternative* to admission" in Cochrane Protocols, Trials 55

#16 "crisis cafe*" or "crisis pad*" 0

#17 "acute day unit*" or "acute day hospital*" or "acute day treatment*" in Cochrane Protocols, Trials 10

#18 MeSH descriptor: [Personality Disorders] explode all trees 1460

#19 MeSH descriptor: [Crisis Intervention] explode all trees 210

#20 MeSH descriptor: [Hospitals, Psychiatric] explode all trees 249

#21 MeSH descriptor: [Psychiatric Department, Hospital] explode all trees 101

#22 MeSH descriptor: [Emergency Services, Psychiatric] explode all trees 50

#23 (#1 OR #2 OR #3 OR #4 OR #5 OR #6 OR #7 OR #8 OR #9 OR #10 OR #11 OR #18) AND (#12 OR #13 OR #14 OR #15 OR #16 OR #17 OR #19 OR #20 OR #21 OR #22) in Cochrane Protocols, Trials 62

---------------------

**Database 4:** WEB OF SCIENCE

Date parameters: 1900 until February 24^th^ 2022

Date of search: 2^nd^ March 2022

**"personality disorder" OR "complex emotional needs" OR ("complex needs" AND ("mental health" or emotion*)) OR "emotion* dysreg*" OR "personality difficult*" OR "personality dysfunction*" OR "affect regulation disorder" OR "emotion* instab*" OR "Cluster A personalit*" OR "Cluster B personalit*" OR "borderline personalit*" OR "emotion* unstable personalit* disorder" OR "impulsive personalit*" OR "histrionic personalit*" OR "narcissistic personalit*" OR "antisocial personalit*" OR "dissocial personalit*" OR "paranoid personalit*" OR "schizoid personalit*" OR "schizotypal personalit*" OR "avoidant personalit*" OR "anxious personalit*" OR "dependent personalit*" OR "obsessive compulsive personalit*" OR "anankastic personalit*" OR "sociopathic personalit*" OR "mixed personality disorder*"** (Topic) and **"crisis intervention" OR "crisis service*" OR "crisis care" OR "emergency psychiatric services" OR "brief intervention" OR "crisis team*" OR "home treatment team*" OR "crisis house" OR "recovery house" OR "sanctuar*" OR "safe haven" OR "alternative* to hosp*" OR "alternative* to admission*" OR "crisis caf*" or "crisis pad" or ((hospital OR admission OR admitt* OR ward) AND ("mental health" OR psychiatr*)) OR "acute day unit*" or "acute day hospital*" or "acute day treatment*"** (Topic)

**Database 5**: HMIC Health Management Information Consortium via OVID

Date parameters: 1979 and March 2022

Date of search: 2^nd^ March 2022

1 personality disorder.mp. or exp Personality Disorders/

2 "complex emotional need*".mp.

3 ("complex needs" and ("mental health" or "emotion")).mp. [mp=title, other title, abstract, heading words]

4 "emotion* dysreg*".mp. [mp=title, other title, abstract, heading words]

5 "personality difficult*".mp. [mp=title, other title, abstract, heading words]

6 "personality dysfunction*".mp. [mp=title, other title, abstract, heading words]

7 "affect regulation disorder*".mp. [mp=title, other title, abstract, heading words]

8 "emotion* instab*".mp. [mp=title, other title, abstract, heading words]

9 borderline state*.mp. [mp=title, other title, abstract, heading words]

10 borderline state.mp. or exp Borderline States/

11 "Cluster A personalit*".mp. [mp=title, other title, abstract, heading words]

12 "Cluster B personalit*".mp. [mp=title, other title, abstract, heading words]

13 (borderline personalit* or emotion* unstable personalit* disorder or impulsive personalit* or histrionic personalit* or narcissistic personalit* or antisocial personalit* or dissocial personalit* or paranoid personalit* or schizoid personalit* or schizotypal personalit* or avoidant personalit* or anxious personalit* or dependent personalit* or obsessive compulsive personalit* or anankastic personalit* or sociopathic personalit* or mixed personality disorder*).mp. [mp=title, other title, abstract, heading words]

14 crisis intervention.mp. or exp Crisis Intervention/

15 ("crisis service*" or "crisis intervention service*" or "crisis care").mp. [mp=title, other title, abstract, heading words]

16 exp Crisis Intervention Services/

17 exp Psychiatric Hospitals/

18 exp Psychiatric Units/

19 exp Psychiatric Hospital Admission/ or exp Psychiatric Hospitalization/

20 exp Brief Psychotherapy/ or brief intervention.mp.

21 ("crisis team*" or "home treatment team*").mp. [mp=title, other title, abstract, heading words]

22 (Crisis house or recovery house or sanctuar* or "safe haven" or "alternative* to hosp*" or "alternative* to admission").mp. [mp=title, other title, abstract, heading words]

23 (crisis cafe* or crisis pad*).mp. [mp=title, other title, abstract, heading words]

24 ((hospital or admission or admitt* or ward) and (mental health or psychiatr*)).mp. [mp=title, other title, abstract, heading words]

25 ("acute day unit*" or "acute day hospital*" or "acute day treatment*").mp. [mp=title, other title, abstract, heading words]

26 1 or 2 or 3 or 4 or 5 or 6 or 7 or 8 or 9 or 10 or 11 or 12 or 13

27 14 or 15 or 16 or 17 or 18 or 19 or 20 or 21 or 22 or 23 or 24 or 25

28 26 and 27

29 "complex emotional need*".mp.

30 ("complex needs" and ("mental health" or "emotion*")).mp. [mp=title, other title, abstract, heading words]

31 emotion* dysreg*.mp. [mp=title, other title, abstract, heading words]

32 personality difficult*.mp. [mp=title, other title, abstract, heading words]

33 personality dysfunction*.mp. [mp=title, other title, abstract, heading words]

34 affect regulation disorder.mp. [mp=title, other title, abstract, heading words]

35 emotion* instab*.mp. [mp=title, other title, abstract, heading words]

36 borderline state*.mp. [mp=title, other title, abstract, heading words]

37 Cluster A personalit*.mp. [mp=title, other title, abstract, heading words]

38 Cluster B personalit*.mp. [mp=title, other title, abstract, heading words]

39 (borderline personalit* or emotion* unstable personalit* disorder or impulsive personalit* or histrionic personalit* or narcissistic personalit* or antisocial personalit* or dissocial personalit* or paranoid personalit* or schizoid personalit* or schizotypal personalit* or avoidant personalit* or anxious personalit* or dependent personalit* or obsessive compulsive personalit* or anankastic personalit* or sociopathic personalit* or mixed personality disorder*).mp. [mp=title, other title, abstract, heading words]

40 brief intervention*.mp.

41 ("crisis team*" or "home treatment team*").mp. [mp=title, other title, abstract, heading words]

42 (Crisis house or recovery house or sanctuar* or "safe haven" or "alternative* to hosp*" or "alternative* to admission").mp. [mp=title, other title, abstract, heading words]

43 (crisis cafe* or crisis pad*).mp. [mp=title, other title, abstract, heading words]

44 ((hospital or admission or admitt* or ward) and (mental health or psychiatr*)).mp. [mp=title, other title, abstract, heading words]

45 ("acute day unit*" or "acute day hospital*" or "acute day treatment*").mp. [mp=title, other title, abstract, heading words]

46 personality disorder.mp. or exp Personality disorders/

47 "crisis intervention".mp. or exp Crisis intervention/ or "crisis care".mp. or "crisis service*".mp. [mp=title, other title, abstract, heading words]

48 exp Psychiatric emergency services/

49 exp Mental health hospitals/

50 29 or 30 or 31 or 32 or 33 or 34 or 35 or 36 or 37 or 38 or 39 or 46

51 40 or 41 or 42 or 43 or 44 or 45 or 47 or 48 or 49

52 50 and 51

**Database 6**: CINAHL via Ebschohost

Date parameters: 1976 to 25^th^ February 2022

Date of search: 2^nd^ March 2022

| **#** | **Query** |
| --- | --- |
| S50 | S48 AND S49 |
| S49 | S37 OR S38 OR S39 OR S40 OR S41 OR S42 OR S43 OR S44 OR S45 OR S46 OR S47 |
| S48 | S26 OR S27 OR S28 OR S29 OR S30 OR S31 OR S32 OR S33 OR S34 OR S35 OR S36 |
| S47 | ""acute day unit*" or "acute day hospital*" or "acute day treatment*"" |
| S46 | (MH "Hospitals, Psychiatric") OR "psychiatric hospital*" |
| S45 | (hospital or admission or admitt* or ward) AND (mental health or psychiatr*) |
| S44 | crisis cafe* or crisis pad* |
| S43 | "Crisis house" OR "recovery house" OR sanctuar* OR "safe haven" OR "alternative* to hosp*" OR "alternative* to admission"" |
| S42 | ""crisis team*" or "home treatment team*"" OR (MH "Rapid Response Team") |
| S41 | "brief intervention" OR (MH "Psychotherapy, Brief+") |
| S40 | (MH "Psychotherapy, Brief") OR "brief intervention*" |
| S39 | (MH "Emergency Services, Psychiatric") OR (MH "Psychiatric Emergencies") OR "emergency psychiatric service" |
| S38 | "crisis care" OR "crisis service*" OR "crisis intervention service" |
| S37 | (MH "Crisis Intervention") OR "Crisis Intervention" OR (MH "Crisis Therapy (Saba CCC)") OR (MH "Crisis Theory") |
| S36 | borderline personalit* or emotion* unstable personalit* disorder or impulsive personalit* or histrionic personalit* or narcissistic personalit* or antisocial personalit* or dissocial personalit* or paranoid personalit* or schizoid personalit* or schizotypal personalit* or avoidant personalit* or anxious personalit* or dependent personalit* or obsessive compulsive personalit* or anankastic personalit* or sociopathic personalit* or mixed personality disorder* |
| S35 | "Cluster A personalit*" OR "Cluster B personalit*" |
| S34 | "borderline state*" |
| S33 | "emotion* instab*" |
| S32 | "affect regulation disorder" |
| S31 | "personality dysfunction*" |
| S30 | "personality difficult*" |
| S29 | (MH "Emotional Lability") OR "emotion* dysreg*." OR (MH "Emotional Regulation") |
| S28 | ""complex needs" and ("mental health" or "emotion*")" |
| S27 | ""complex emotional need*"" |
| S26 | "personality disorder" OR (MH "Personality Disorders+") |
| S25 | S23 AND S24 |
| S24 | S12 OR S13 OR S14 OR S15 OR S16 OR S17 OR S18 OR S19 OR S20 OR S21 OR S22 |
| S23 | S1 OR S2 OR S3 OR S4 OR S5 OR S6 OR S7 OR S8 OR S9 OR S10 OR S11 |
| S22 | ""acute day unit*" or "acute day hospital*" or "acute day treatment*"" |
| S21 | (MH "Hospitals, Psychiatric") OR "psychiatric hospital*" |
| S20 | (hospital or admission or admitt* or ward) AND (mental health or psychiatr*) |
| S19 | crisis cafe* or crisis pad* |
| S18 | "Crisis house" OR "recovery house" OR sanctuar* OR "safe haven" OR "alternative* to hosp*" OR "alternative* to admission"" |
| S17 | ""crisis team*" or "home treatment team*"" OR (MH "Rapid Response Team") |
| S16 | "brief intervention" OR (MH "Psychotherapy, Brief+") |
| S15 | (MH "Psychotherapy, Brief") OR "brief intervention*" |
| S14 | (MH "Emergency Services, Psychiatric") OR (MH "Psychiatric Emergencies") OR "emergency psychiatric service" |
| S13 | "crisis care" OR "crisis service*" OR "crisis intervention service" |
| S12 | (MH "Crisis Intervention") OR "Crisis Intervention" OR (MH "Crisis Therapy (Saba CCC)") OR (MH "Crisis Theory") |
| S11 | borderline personalit* or emotion* unstable personalit* disorder or impulsive personalit* or histrionic personalit* or narcissistic personalit* or antisocial personalit* or dissocial personalit* or paranoid personalit* or schizoid personalit* or schizotypal personalit* or avoidant personalit* or anxious personalit* or dependent personalit* or obsessive compulsive personalit* or anankastic personalit* or sociopathic personalit* or mixed personality disorder* |
| S10 | "Cluster A personalit*" OR "Cluster B personalit*" |
| S9 | "borderline state*" |
| S8 | "emotion* instab*" |
| S7 | "affect regulation disorder" |
| S6 | "personality dysfunction*" |
| S5 | "personality difficult*" |
| S4 | (MH "Emotional Lability") OR "emotion* dysreg*." OR (MH "Emotional Regulation") |
| S3 | ""complex needs" and ("mental health" or "emotion*")" |
| S2 | ""complex emotional need*"" |
| S1 | "personality disorder" OR (MH "Personality Disorders+") |

**Database 7:** APA PsycExtra

Date parameters: 1908 to March 1st 2022

Date of search: 2^nd^ March 2022

1 "complex emotional need*".mp.

2 ("complex needs" and ("mental health" or "emotion*")).mp. [mp=title, abstract, heading word, keywords]

3 emotion* dysreg*.mp. [mp=title, abstract, heading word, keywords]

4 personality difficult*.mp. [mp=title, abstract, heading word, keywords]

5 personality dysfunction*.mp. [mp=title, abstract, heading word, keywords]

6 affect regulation disorder.mp. [mp=title, abstract, heading word, keywors]

7 emotion* instab*.mp. [mp=title, abstract, heading word, keywords]

8 borderline state*.mp. [mp=title, abstract, heading word, keywords]

9 Cluster A personalit*.mp. [mp=title, abstract, heading word, keywords]

10 Cluster B personalit*.mp. [mp=title, abstract, heading word, keywords]

11 (borderline personalit* or emotion* unstable personalit* disorder or impulsive personalit* or histrionic personalit* or narcissistic personalit* or antisocial personalit* or dissocial personalit* or paranoid personalit* or schizoid personalit* or schizotypal personalit* or avoidant personalit* or anxious personalit* or dependent personalit* or obsessive compulsive personalit* or anankastic personalit* or sociopathic personalit* or mixed personality disorder*).mp. [mp=title, abstract, heading word, keywords]

12 brief intervention*.mp.

13 ("crisis team*" or "home treatment team*").mp. [mp=title, abstract, heading word, keywords]

14 (Crisis house or recovery house or sanctuar* or "safe haven" or "alternative* to hosp*" or "alternative* to admission").mp. [mp=title, abstract, heading word, keywords]

15 (crisis cafe* or crisis pad*).mp. [mp=title, abstract, heading word, keywords]

16 ((hospital or admission or admitt* or ward) and (mental health or psychiatr*)).mp. [mp=title, abstract, heading word, keywords]

17 ("acute day unit*" or "acute day hospital*" or "acute day treatment*").mp. [mp=title, abstract, heading word, keywords]

18 ("crisis service*" or "crisis care").mp. [mp=title, abstract, heading word, keywords]

19 crisis intervention.mp. or exp Crisis Intervention/

20 personality disorder.mp. or exp Personality Disorders/

21 emergency service psychiatric.mp.

22 exp Psychiatric Hospitals/

23 1 or 2 or 3 or 4 or 5 or 6 or 7 or 8 or 9 or 10 or 11 or 20

24 12 or 13 or 14 or 15 or 16 or 17 or 19 or 21 or 22

25 23 and 24

**Database 8:** Embase

Date parameters: 1974 to 2022 Week 08

Date of search: 2^nd^ March 2022

1 "personality disorder".mp. or exp personality disorder/

2 "complex emotional need*".mp.

3 ("complex needs" and ("mental health" or "emotion*")).mp. [mp=title, abstract, heading word, drug trade name, original title, device manufacturer, drug manufacturer, device trade name, keyword heading word, floating subheading word, candidate term word]

4 "emotion* dysreg*".mp.

5 "personality difficult*".mp.

6 "personality dysfunction*".mp.

7 "emotion* instab*".mp.

8 exp borderline state/ or "borderline state*".mp.

9 "Cluster A personalit*".mp.

10 "Cluster B personalit*".mp.

11 ("borderline personalit*" or "emotion* unstable personalit* disorder" or "impulsive personalit*" or "histrionic personalit*" or "narcissistic personalit*" or "antisocial personalit*" or "dissocial personalit*" or "paranoid personalit*" or "schizoid personalit*" or "schizotypal personalit*" or "avoidant personalit*" or "anxious personalit*" or "dependent personalit*" or "obsessive compulsive personalit*" or "anankastic personalit*" or "sociopathic personalit*" or "mixed personality disorder*").mp. [mp=title, abstract, heading word, drug trade name, original title, device manufacturer, drug manufacturer, device trade name, keyword heading word, floating subheading word, candidate term word]

12 "crisis intervention".mp. or exp crisis intervention/

13 exp psychiatric emergency/

14 "brief intervention".mp.

15 ("crisis team*" or "home treatment team").mp. [mp=title, abstract, heading word, drug trade name, original title, device manufacturer, drug manufacturer, device trade name, keyword heading word, floating subheading word, candidate term word]

16 ("Crisis house*" or "recovery house*" or sanctuar* or "safe haven" or "alternative* to hosp*" or "alternative* to admission").mp. [mp=title, abstract, heading word, drug trade name, original title, device manufacturer, drug manufacturer, device trade name, keyword heading word, floating subheading word, candidate term word]

17 ("crisis cafe*" or "crisis pad*").mp. [mp=title, abstract, heading word, drug trade name, original title, device manufacturer, drug manufacturer, device trade name, keyword heading word, floating subheading word, candidate term word]

18 ("acute day unit*" or "acute day hospital*" or "acute day treatment*").mp. [mp=title, abstract, heading word, drug trade name, original title, device manufacturer, drug manufacturer, device trade name, keyword heading word, floating subheading word, candidate term word]

19 ((hospital or admission or admitt* or ward) and ("mental health" or psychiatr*)).mp. [mp=title, abstract, heading word, drug trade name, original title, device manufacturer, drug manufacturer, device trade name, keyword heading word, floating subheading word, candidate term word]

20 exp mental hospital/

21 1 or 2 or 3 or 4 or 5 or 6 or 7 or 8 or 9 or 10 or 11

22 12 or 13 or 14 or 15 or 16 or 17 or 18 or 19 or 20

23 21 and 22

24 1 or 2 or 3 or 4 or 5 or 6 or 7 or 9 or 10 or 11

25 22 and 24
