# Supplementary material for: Crisis and acute mental health care for people who have been given a diagnosis of a ‘personality disorder’: a systematic review
Source: BMC Psychiatry. 2023 Oct 5;23:720. doi: 10.1186/s12888-023-05119-7 (PMC10552436; doi:10.1186/s12888-023-05119-7)
Supplement: Supplementary file 2 — Additional file 2: Table S1. Assessment of the quality of studies reporting pre and post intervention outcomes over time, without a comparison group, using the Newcastle Ottowa Scale. Table S2. Outcomes: hospital admission. Table S3. Outcomes: brief admission. Table S4. Outcomes: Crisis teams. Table S5. Outcomes: Acute Day Unit. Table S6. Outcomes: Crisis-focused psychotherapies or psychosocial interventions. Table S7. Outcomes: Crisis-focused psychotherapies or psychosocial interventions In emergency departments or psychiatric emergency services. Table S8. Outcomes: Crisis-focused psychotherapies or psychosocial interventions. Table S9. Outcomes: Mother and Baby Units. Table S10. Outcomes: Joint crisis plans. Table S11. Outcomes: Early Intervention Service. [file 12888_2023_5119_MOESM2_ESM.docx]

S2: Supplementary tables:

Table S1: Assessment of the quality of studies reporting pre and post intervention outcomes over time, without a comparison group, using the Newcastle Ottowa Scale

|  | Selection | | | | Comparability | Outcome | | | |
| --- | --- | --- | --- | --- | --- | --- | --- | --- | --- |
| Study | Representativeness | Selection non exposed | Ascertainment of exposure | Outcome of interest not present at start | Comparability | Assessment of outcome | Follow up time | Adequacy of follow up | Total score |
| Eckerstrom | * |  | * |  |  |  |  | * | 3 |
| Barbarato | * |  | * |  |  | * | * |  | 4 |
| Nehls |  |  | * |  |  | * | * | * | 4 |
| Turhan | * |  | * | * |  | * | * | * | 6 |
| Savard | * |  | * | * |  |  | * |  | 4 |
| Yen |  |  | * | * | * |  | * | * | 5 |
| Shergill | * |  | * | * |  |  | * | * | 5 |
| Lariviere | * |  | * | * |  |  | * |  | 4 |
| Vasquez Bourgon |  |  | * | * |  |  | * | * | 4 |
| Huxley |  |  | * | * |  |  | * | * | 4 |
| McQuillian | * |  | * | * |  |  | * | * | 5 |
| Pavan | * |  | * | * |  |  | * | * | 5 |
| Booth |  |  | * | * |  |  | * | * | 4 |
| Breslow | * |  | * | * |  |  | * | * | 5 |
| Wright | * |  | * | * |  |  | * | * | 5 |
| Gerbardht |  |  | * | * |  |  | * | * | 4 |
| Unger | * |  | * | * |  |  | * | * | 5 |
| Mellsop | * |  | * | * |  |  | * | * | 5 |
| Branjerdporn | * |  | * | * |  | * | * | * | 6 |
| Yoshimatsu | * |  | * | * |  |  | * | * | 5 |
| Giese | * |  | * | * |  |  | * | * | 5 |
| Koekkoek | * |  | * | * |  | * | * | * | 6 |

Table S1. Assessment of the quality of studies reporting pre and post intervention outcomes, using the Newcastle Ottowa Scale. 1 star contributes 1 point towards the total score. The higher the score the lower the risk of bias.

*Results tables by outcome:*

|  | **Table S2. Hospital admission** | | | | | | | | | | | |
| --- | --- | --- | --- | --- | --- | --- | --- | --- | --- | --- | --- | --- |
| **Outcome** | | **Outcome measure** | **Author** | **Outcome timepoint** | | | | **Outcome** | | | **Statistical test** | **Additional results** |
|  | | | | Admission | 7 days | Discharge | | | | 12 months |  | |
| Symptomatic Improvement | | Beck Depression Inventory | Unger | 29.94 (10.36) |  | 15.37 (11.21) | | | | 19.16 (13.32) | Cohen’s d:  T1-T2 1.35, T2-T3 0.31 | Recovery rate:  T1-2 38.8%  T2-T3 29.9% |
|  |  |  | Giese | 29.7 | 21.8 | 17 | | | |  | Repeated measures analysis of improvement over time p<0.05 |  |
|  |  | HRSD (mean) | Giese | 24.2 | 16.6 | 12.2 | | | |  |  |  |
|  |  |  | Unger | 29.48 (6.39) |  | 7.90 (7.35) | | | | 10.87 (8.25) | Cohen’s d T1-2 3.13 T2-3 0.38 p<0.001 | Recovery rate 66.7% |
|  |  | HAS | Giese | 21.9 | 16.6 | 12.2 | | | |  |  |  |
|  |  | GAS | Giese | 36.9 | 48.8 | 56 | | | |  |  |  |
|  |  | SCL-90R | Giese | Reports subscales only |  |  | | | |  | Reports significant improvement |  |
|  |  | BPRS | Giese | Reports subscales only |  |  | | | |  | Reports significant improvement |  |
|  |  | BSI-GSI (mean) | Unger | 1.67 (0.65) |  | 0.81 (0.65) | | | | 1.14 (0.78) | Cohen’s d T1-2 1.34 T2-3 0.42 p<0.001 | Recovery rate 46.30% |
|  |  | CGI (median) | Unger | 5 |  | 4 | | | |  | Cohen’s d 0.79 p<0.001 |  |
|  |  |  | Gebhardt | 6 (0.6) |  | 4.5 (1.2) | | | |  |  |  |
|  |  | GAF (median) | Unger | 45 |  | 65 | | | |  | Cohen’s d 0.81 p<0.001 |  |
|  |  |  | Gebhardt | 46.1 (10.2) |  | 64.4 (11.6) | | | |  | Change 18.3 (14.7) p=0.026 |  |
|  | | | | Before intervention: | | Post intervention | | | | |  | |
|  | |  |  | Pre | Post | | Pre | | | Post |  |  |
|  |  | BSI-GSI (mean) | Uhlmann | 77.0 (8.48) | 74.1 (7.83) | | 78.9 (2.66) | | | 77.4 (3.59) | Cohen’s d=0.28 before and 0.49 after intervention. Within group: p<0.025  No sign between group differences |  |
|  | |  |  | Admission | | | | | Discharge | | | Change |
|  | | HAMD | Yoshimatsu | 34.1 (6.46) | | | | | 12.0 (6.80) | | |  |
| HoNOS | | HoNOS | Branjerdporn | 20.38 (7.22) | | | | | 7.79 (6.61) | | | -12.78 (10.77) |
|  | | | | Pre admission | | | | | 6 months after admission | | |  |
| Adaptive functioning | | Axis V | Mellsop | 4.30 (0.89) | | | | | 3.65 (1.33) | | |  |
|  | | SAS-M | Mellsop | 2.33 (0.44) | | | | | 3.65 (1.33) | | |  |
|  | | GHQ-9 | Mellsop |  | | | | | 12.95 (17.4) | | |  |
|  | | CGHQ | Mellsop |  | | | | | 11.65 (8.62) | | |  |
| Table S2. Summary of outcome data reported following hospital admission.  *HAS= Hamilton Rating Scale for Anxiety GAS= Global Assessment Scale SCL-90R= Revised Symptom Checklist BPRS= Brief Psychiatric rating scale. HRSD= Hamilton Rating Scale for Depression GAF= Global Assessment of Functioning CGI= Clinical Global Impression Scale BSI-GSI- Brief Symptom Inventory HoNOS= health of the nation outcome score GHQ-9 General Health Questionnaire SAS-M Social Adjustment Scale-Modified CGHQ- clinical general health questionnaire HAMD=* | | | | | | | | | | | | |

| **Table S3. Brief admission** | | | | | | | | |
| --- | --- | --- | --- | --- | --- | --- | --- | --- |
| Symptomatic improvement | **Outcome measure** | **Author** | **Timepoint** | **Outcome** | | **Statistical tests** | | |
|  | BPRS | Barbato | From admission to discharge | Change in score:  9.2 | | Cohen’s d 0.81 p<0.001 | | |
|  | HADS | Eckerstrom | From admission to discharge  1^st^ admission  2^nd^ admission  3^rd^ admission  4^th^ admission | Change in score:    -14.9  -11.4  -14.3  -15.1 | | t 5.84 p<0.001  t 4.06 p<0.001  t 3.96 p<0.001  t 3.87 p=0.001 | | |
|  | | | Pre | Post | |  | | |
| Service use | Mean days in hospital over 6 months | Koekkoek | 41.8 | 29.5 | | P=0.53 | | |
|  |  | Westling | Intervention:  59.95 (49.88)    Control:  51.55 (39.53) | Intervention:  45.45 (50.08)    Control:  40.56 (48.21) | | See results below for 12 month outcome | | |
|  | Mean days in hospital over 12 months | Van Kessel | Intervention:  106 (85)    Control:  49 (34) | Intervention: 27 (33)    Control:  58 (87) | | Within group:  P<0.005      No significant difference | | Time x unit interaction F(1,19) =7.5 p<0.02 |
|  |  | Westling |  | Intervention:  30.14 (44.98)    Control:  29.44 (40.61) | | P<0.001        P<0.001 | | LGCM -4.06 p=0.33 |
| Health related QOL | EQ-5D | Eckerstrom | Change in score:  1^st^ admission: 44.7%  2^nd^ admission: 38.9%  3^rd^ admission: 46.9%  4^th^ admission: 40.5% | | | T -4.98 p<0.001  T -3.02 p=0.004  T -2.80 p=0.009  T -3.08 p=0.005 | | |
|  | | | Pre | 6 months | 12 months |  | | |
| NSSI and suicide attempts | Mean number of NSSI events within 2 weeks of timepoint | Westling | Intervention:  6.84 (7.62) | Intervention:  4.86 (7.62) | Intervention:  4.41 (6.58) | Within group:  P=0.047    P=0.59 | Between group:  LGCM -0.41 p=0.09 | |
|  |  |  | Control:  5.44 (8.18) | Control:  5.15 (7.00) | Control:  4.44 (6.11) |  |  |  |
|  |  |  | Pre | Post | |  | | |
| Therapeutic alliance | Clinician rated agreement with treatment | Koekkoek | 3.8 | 6.2 | | P=0.02 | | |
| Table S3. Summary of outcome data reported following brief admission. *EQ-5D= EuroQol 5 dimensions. NSSI= non-suicidal self injury. BPRS= Brief Psychiatric Rating Scale HADS= Hospital Anxiety and Depression Scale* | | | | | | | | |

| **Table S4. Crisis team** | | | | |
| --- | --- | --- | --- | --- |
| **Outcome** | **Outcome measure** | **Author** | **Outcome** | **Statistical test** |
| Hospitalisation |  | Turhan | 34.4% |  |
| Symptomatic improvement | CGI | Turhan | Improved (scored 1 or 2): 40.6%    Did not improve (scored 3 or more): 61.3% |  |
| Table S4. Summary of outcome data reported following use of crisis teams *CGI= Clinical Global Impression* | | | | |

| **Table S5. Acute Day Unit** | | | | | | |
| --- | --- | --- | --- | --- | --- | --- |
| **Outcome** | **Outcome measure** | **Author** | **Timepoint** | | **Outcome** | **Statistical test** |
|  |  |  | Pre-admission | | Discharge |  |
| Symptomatic Improvement | OQ-45.2 | Savard | 98.1 (21.87) | | 79.46 (25.83) | Cohen's d 0.78 (CI 0.60-0.98) p<0.001 |
|  | Symptom distress subscale | Savard | 60.15 (13.64) | | 48.42 (16.23) | Cohen's d 0.78 p<0.001 |
|  | Interpersonal relations subscale | Savard | 21.26 (6.51) | | 17.79 (6.78) | Cohen's d 0.52 (0.35-0.70) p<0.001 |
|  | Social role subscale | Savard | 16.34 (5.34) | | 13.26 (5.71) | Cohen's d 0.56 (0.38-0.73) p<0.001 |
|  | Modified remission from depression questionnaire | Zimmerman | In person:  35.0 (7.7)    Telehealth Group:  33.8 (7.4) | | In person:  17.8 (10.4)    Telehealth Group:  18.0 (9.9) | Cohen’s d:  1.88 in person and 1.81 telehealth  Significant difference only on subscales of functioning (improvement telehealth>in person p<0.05) and anger (Improvement in person>telehealth p<0.05) |
|  | SCL90-R (participant) | Lariviere | 2.1 (0.6) | | 0.9 (0.8) | p<0.001 |
|  | SCL90-R | Lariviere | 4.7 (1.6) | | 2.4 (1.5) | P=0.001 |
|  | Perceived distress | Lariviere | 2.7 (0.6) | | 1.8 (0.7) | P<0.001 |
|  |  |  | Discharge | | 3 months |  |
|  | BDI | Yen | 33.5 (12.4) | | 27.3 (14.8) | Cohen's d 0.45 p<0.05 |
|  | Beck Hopelessness scale | Yen | 13.0 (5.5) | | 10.0 (6.7) | Cohen's d 0.49 p<0.01 |
|  | Dissociative Experiences Scale | Yen | 26.7 (18.9) | | 20.4 (16.6) | Cohen's d 0.35 p<0.01 |
|  | STAXI | Yen | 94.3 (22.4) | | 88.2 (26.9) | Cohen's d 0.25 p<0.05 |
|  | BSI | Yen | 2.1 (0.7) | | 1.8 (0.8) | Cohen's d 0.40 p<0.05 |
|  |  | | Mean difference admission to discharge (SD) | | |  |
|  | BPRS | Vazquez-Bourgon | 9.7 (6.2) | | |  |
|  | CGI | Vazquez-Bourgon | 0.8 (0.8) | | |  |
|  | HoNOS | Vazquez-Bourgon | 3.9 (2.7) | | |  |
|  | HoNOS social subscale | Vazquez-Bourgon | 0.5 (0.8) | | |  |
|  | | | Admission | Discharge | |  |
| Social participation | sa Scale- total | Lariviere | 6.6 (1.0) | 7.5 (1.0) | | P=0.003 |
|  | Satisfaction with social participation | Lariviere | 2.5 (0.5) | 3.1 (0.6) | | P=0.001 |
| Patient satisfaction | CUPPS questionnaire | Zimmerman | In person group: 100% indicated very or extremely satisfied  Telehealth: 95.4% indicated very or extremely satisfied | | |  |
| Table S5. Outcome data reported following use of acute day units *OQ-45.2=Outcome Questionnaire SCL-90R= Revised Symptom Checklist BDI= Beck Depression Inventory BSI=Brief Symptom Inventory CGI= Clinical Global Impression BPRS= Brief Psychiatric Rating Scale HoNOS= Health of the Nation Outcome Score STAXI= State-Trait Anger Expression Inventory* | | | | | | |

| **Table S6. Crisis-focused psychotherapies or psychosocial interventions**  **Outpatient based:** | | | | | |
| --- | --- | --- | --- | --- | --- |
| **Outcome** | **Outcome measure** | **Author** | **Timepoint** | **Outcome** | **Statistical test** |
| Hospitalisation | % hospitalised | Andreoli | 3 months | Intervention 1: AP-P 14.3%  Intervention 2: AP-N 14.3%  TAU: 36.7% | AP-P vs TAU  Chi2 6.34 p=0.012  APN-N vs TAU  Chi2 6.34 p=0.012   No difference between AP-P and AP-N |
|  |  | McQuillian | By end of treatment | 6% |  |
|  | Time to hospitalisation | Andreoli |  |  | AP-P vs TAU Chi2 6.39 p=0.12  AP-N vs TAU  Chi2 6.88 p=0.009  No difference between AP-P and AP-N |
|  |  |  | Baseline (over 18 months) | Active phase (over 18 months) |  |
|  | Mean number of inpatient admissions | Grenyer | Intervention:  1.62(1.23)  TAU:  1.4 (1.76) | Intervention:  0.5 (1.21)  TAU:  0.60 (1.71) | Time site interaction 0.006 p=0.937 |
|  | Mean bed days | Grenyer | Intervention:  13.46 (26.62)  TAU:  12.98 (23.78) | Intervention:  12.98 (23.78)  TAU:  8.44 (29.86) | Time site interaction 4.301 p=0.038 |
|  | | | Timepoint | Outcome |  |
| Suicide attempt or suicide | Mean number of suicidal relapses | Andreoli | 3 months | Intervention 1: AP-P vs TAU    Intervention 2: AP-N vs TAU | Chi2 8.09 p=0.004      Chi2 9.33 p=0.002 |
|  | Time to suicidal relapse | Andreoli | NA | Intervention 1: AP-P vs TAU    Intervention 2: AP-N vs TAU | Chi2 7.63 p=0.006      Chi2 9.87 p=0.002 |
|  | | | Baseline | End of treatment |  |
| Symptomatic improvement | DSM-5 symptoms | Huxley | 7.55 (1.62) | 6.43 (2.21) | Cohen’s d 0.58  p<0.001 |
|  | Mental health inventory | Huxley | 21.28 (4.69) | 17.40 (5.18) | D 0.79 p<0.001 |
|  | BDI item on suicidal ideation | Huxley | 2.30 (0.75) | 1.56 (0.7) | D 1.01 p<0.001 |
|  | QoL single question | Huxley | 37.34 (18.45) | 44.63 (19.99) | D 0.95 p<0.001 |
|  | BHS | McQuillian | 11.0 (4.7) | 9.7 (5.3) | D 0.26 p=0.012 |
|  | SASS | McQuillian | 31.3 (7.0) | 32.7 (7.8) | D 0.23 p=0.152 |
|  |  | Pavan | 38.86 (5.79) | 43.00 (6.75) | D 0.66 p=0.0001 |
|  | BDI | McQuillian | 29.7 (10.9) | 22.8 (12.2 | D 0.6 p=0.001 |
|  |  | Pavan | 20.10 (12.63) | 9.59 (7.93) | D 1.02 p=0.0001 |
|  | STAI/state | Pavan | 54.64 (12.32) | 41.38 (10.69) | D 1.1 p=0.0001 |
|  | STAI/trait | Pavan | 51.89 (12.84) | 44.47 (10.75) | D 0.62 p=0.002 |
|  | STAXI/state | Pavan | 14.44 (5.17) | 11.56 (2.64) | D 0.73 p=0.006 |
|  | STAXI/trait | Pavan | 20.85 (6.62) | 17.94 (5.35) | D 0.48 P=0.0001 |
|  | HDRS | Pavan | 16.18 (6.2) | 6.85 (4.41) | D 1.75 P=0.01 |
|  | GAS | Pavan | 66.9 (8.05) | 80.90 (8.99) | D 1.64 P=0.05 |
| Table S6. Outcome data reported following use of outpatient based psychological or psychosocial interventions.  *BDI= Beck depression Inventory BHS= Beck Hopelessness Scale SASS= Social Adaption Self-Evaluation Scale QoL= Quality of Life STAXI= State-Trait Anger Expression Inventory HDRS= Hamilton Depression Rating Scale GAS= Global Assessment Scale* | | | | | |

| **Table S7. Crisis-focused psychotherapies or psychosocial interventions**  **In emergency departments or psychiatric emergency services** | | | | | |
| --- | --- | --- | --- | --- | --- |
| **Outcome** | **Outcome measure** | **Author** | **Timepoint** | **Outcome** | **Statistical test** |
| Repeat suicide attempt | % who had made attempt | Berrino | 3 months | Intervention: 8% Control group 17% | P=0.05 |
|  | Time to repeat attempt (days) | Berrino | NA | Intervention: 85.6 (16.3)  Control:  79.8 (25.9) | P=0.05 |
| Hospitalisation | Time to readmission (days) | Berrino | NA | Intervention: 81.1 (23.6)  Control: 42.2 (43.6) | P<0.001 |
|  | Days of hospitalisation | Berrino | 3 months | Intervention: 1.94 (7.79)  Control: 9.3 (16.5) | P<0.001 |
|  | | | Before intervention | After intervention |  |
|  | Rate of hospitalisation | Damsa 2003 | 18.50% | 7.90% | P<0.001 |
|  |  | Damsa 2005 | 32.20% | 15% | P=0.005 |
|  |  | | Admission | Discharge |  |
| Symptomatic improvement | PSAS | Breslow  (study replicated 1 year apart) | Study 1:  20.4  Study 2: 15.98 | Study 1: 15.1    Study 2: 11.34 | Study 1: t= 2.96 p<0.01  Study 2: t=2.99 p<0.01 |
|  | BPRS | Laddis | Intervention:  34.8 (9.7)  Control:  26.8 (8) | Intervention:  14.3 (8.2)  Control:  23 (7.9) | Intervention: p<0.001  Control: no sign within group change  Between group p<0.001 |
|  | BSI | Laddis | Does not report baseline measures | Intervention:  74.2  Control:  84.1 | Between group difference p=0.314 |
|  | Client observation | Laddis | Intervention: 19.7 (4.2)    Control: 12.8 (3.6) | Intervention: 7 (4.8)    Control: 9.0 (3.2) | Within group:  P<0.001    Within group: no sign change  Between groups p=0.001 |
|  | Client self observation | Laddis | Intervention:  32.2 (6.8)    Control:  35.7 (6.2) | Intervention 19.3 (6.8)    Control:  24.7 (5.0) | Within group change p<0.05    No sign without group change  Between group p=0.016 |
| Satisfaction | 5 point satisfaction scale | Breslow | 84% found the intervention helpful or very helpful  2% reported unhelpful 13% neutral position | |  |
| Medication Patterns | % of people with  medication change from baseline | Laddis | Intervention:  41%    Control:  92% | |  |
| Table S7. Outcome data reported following use of psychotherapies or psychosocial interventions in Emergency Departments (ED) or Psychiatric Emergency Services (PES). *BPRS= Brief Psychiatric Rating scale BSI= Brief Symptom Inventory PSAS= Psychiatric Symptom Assessment Scale* | | | | | |

| **Table S8. Crisis-focused psychotherapies or psychosocial interventions**  **Inpatient groups:** | | | | | | | | |
| --- | --- | --- | --- | --- | --- | --- | --- | --- |
| **Outcome** | **Outcome measure** | **Author** | **Outcome and timepoint** | | | | | **Statistical test** |
|  |  |  | Baseline | | Post group | | 3 months |  |
| Self harm | Frequency of self-harm in previous 6 weeks | Booth | 13.68 (21.81) | | 4.50 (11.01) | | 3.62 (11.3) | T1-T2 p=0.02  T3-T1 p=0.01  ANOVA for time p=0.01 |
|  |  |  | Change score | | | | |  |
| Symptomatic improvement | BDI | Springer | Intervention: -15.5 (3.5)  Control: -15.3 (3.0) | | | | | No significant difference between groups |
|  | Hopelessness (HS) | Springer | Intervention: -5.5 (2)  Control: -5.4 (2.2) | | | | | No significant difference between groups |
|  | Suicidal ideation (ASIQ) | Springer | Intervention: -27.0 (12.5)  Control: -21.0 (10.7) | | | | | No significant difference between groups |
|  | Coping skills (CCQ) | Springer | \| Intervention \| 15.2 (7.1) \| \| --- \| --- \| \| Control \| 24.3 (8) \| | | | | | No significant difference between groups |
|  | Anger expression (STAXI global) | Springer | \| Intervention \| -1.6 (1.6) \| \| --- \| --- \| \| Control \| -4.3 (2.2) \| | | | | | No significant difference between groups |
|  | Locus of control | Springer | Intervention -0.3 (0.7)  Control -1.1 (1.0) | | | | | ACOVA P<0.01 |
|  | | | Baseline | Post group | | 3 months | |  |
| Distress Tolerance | Distress Tolerance Scale | Booth | 6.31 (1.87) | 10.01 (3.35) | | 9.29 (4.676) | | T1-2 p<0.001  T1-T3 p<0.001  T2-T3 no difference  ANOVA for time P<0.001 |
| Table S8. Outcome data reported following use of inpatient psychotherapeutic groups. *BDI=* *Beck Depression Inventory HS= Hopelessness Scale ASIQ= Adult Suicidal Ideation Questionnaire STAXI- Strate-Trait Anger Expression Inventory, CCQ= Creative Coping Questionnaire* | | | | | | | | |

| **Table S9. Mother and Baby Units:** | | | | |
| --- | --- | --- | --- | --- |
| **Outcome** | **Outcome measure** | **Author** | **Outcome and timepoint** | **Statistical test** |
|  |  |  | Change discharge to 3 months |  |
| Global Assessment of Function | GAF | Wright | -6.3 (19.7) | Those with a  personality disorder diagnosis had the lowest functioning at discharge p=0.05 |
| Mother infant relationship | CARE index sensitivity | Wright | -1.2 (2.5) | Those with a personality disorder diagnosis showed a deterioration whereas those without did not (p<0.005) |
| Table S9. Outcome data reported following use of Mother and Baby Units (MBUs). *GAF= Global Assessment of Function CARE= Child and Adult Relational Experimental Index* | | | | |

| **Table S10. Joint crisis plans** | | | | | |
| --- | --- | --- | --- | --- | --- |
| **Outcome** | **Outcome measure** | **Author** | **Outcome and timepoint** | | **Statistical test** |
|  | | | Baseline | 6 months |  |
|  | WAI-C | Borschmann (crisis planning) | Intervention: 58.47 (18.5)  Control: 63.36 (17.92) | 58.85 (16.75)  60.47 (15.92) | No significant between group difference |
|  | WAI-T | Borschmann | Intervention: 63.68 (8.72)  Control: 61.27 (11.10) | 64.44 (10.87)  62.96 (10.74) | No significant between group difference |
|  | CSQ | Borschmann | Intervention: 19.85 (1.46)  Control: 18.62 (1.53) | 19.97 (2.0)  19.64 (1.33) | No significant between group difference |
|  | WEMWBS | Borschmann | Intervention: 29.65 (11.09)  Control: 31.74 (10.14) | 34.33 (11.40)  35.26 (10.26) | No significant between group difference |
|  | WSAS | Borschmann | Intervention: 27.02 (6.46)  Control: 26.95 (7.36) | 25.81 (8.94)  26.06 (7.98) | No significant between group difference |
|  | TES | Borschmann | Intervention: 17.04 (2.97)  Control: 16.52 (2.75) | 25.81 (8.94)  16.0 (3.07) | No significant between group difference |
|  | HADS-D | Borschmann | Intervention: 14.46 (4.07)  Control: 14.48 (5.55) | 14.57 (3.83)  12.94 (4.55) | No significant between group difference |
|  | HADS-A | Borschmann | Intervention: 14.46 (4.07)  Control: 14.48 (5.55) | 14.57 (3.83)  12.94 (4.55) | No significant between group difference |
|  | SES | Borschmann | Intervention: 9.82 (6.04)  Control: 10.41 (7.14) | 8.63 (6.11)  10.88 (5.62) | No significant between group difference |
| Evidence of self-harm behaviours | % who had self harmed over 6 months | Borschmann | Intervention: 100%  Control: 100% | 89.70%  55.60% | No significant between group difference |
|  | Mean number of episodes of self harm over 6 months | Borschmann | Intervention: 51.2 (126.4)  Control 56.2 (102.2) | 20.6 (89.7)  20.3 (67.0) | OR of self harm in comparison tih TAU 1.86 (0.53-6.51) p=0.33  RR 0.74 (0.34-1.63) p=0.46 |
| Table S10. Outcome data reported following use f joint crisis plans *WAI= Working Alliance Inventory CSQ= Client Satisfaction Questionnaire SES= Social Engagement Scale WEMWBS= Warwick-Edinburgh Mental Wellbeing Scale WSAS= Work and Social Adjustment Scale TES= Treatment Experience Scale HADS= Hospital Anxiety and Depression Scale* | | | | | |

| **Table S11. Early Intervention Service** | | | | | |
| --- | --- | --- | --- | --- | --- |
| **Outcome** | **Outcome measure** | **Author** | **Outcome and timepoint** | | **Statistical test** |
| Social function scores | Social Function Questionnaire (SFQ) | Tyrer (early intervention vs TAU) | Those with a PD diagnosis allocated to EIS showed little change, those allocated to hospital improved. | | Service/diagnosis interaction F 6.0 p<0.001. |
|  | | | Baseline | 12 weeks |  |
| Symptomatic improvement | CPRS** | Tyrer | Intervention: 33  Control: 32 | 24  25 |  |
|  | MADRS** | Tyrer | Intervention: 23  Control: 23 | 18  18 |  |
|  | BAS** | Tyrer | Intervention: 22  Control: 17 | 17  16 |  |
| Table S11. Outcome data reported following use of an early intervention service. *CPRS= Comprehensive Psychopathological Rating Scale MADRS= Montgomery and Asberg Depression Rating Scale BAS= Brief Scale for Anxiety HADS-A.* | | | | | |
