# Supplementary material for: Crisis and acute mental health care for people who have been given a diagnosis of a ‘personality disorder’: a systematic review
Source: BMC Psychiatry. 2023 Oct 5;23:720. doi: 10.1186/s12888-023-05119-7 (PMC10552436; doi:10.1186/s12888-023-05119-7)
Supplement: Supplementary file 3 — Additional file 3: Supplementary file 3. GRADE scoring criteria for interventional studies evaluating crisis and acute mental health care for people with complex emotional needs who may have a diagnosis of a ‘personality disorder’. [file 12888_2023_5119_MOESM3_ESM.docx]

**Supplementary file 3: GRADE scoring criteria for interventional studies evaluating** **crisis and acute mental health care for people with complex emotional needs who may have a diagnosis of a ‘personality disorder’**

1.        Study quality / risk of bias: Certainty was downgraded if >50% of studies were considered to be of moderate, serious or high risk of bias.

2.        Inconsistency: Rating was downgraded if there was inconsistency in the direction of change across studies. Rating was not downgraded if most studies reported change in the same direction or where there was only one contributing study and so it was not possible to determine inconsistency.

3.        Indirectness: A judgement was made about whether the study primarily included a population and an intervention of interest for the research question. For example, studies that primarily investigated a crisis intervention in a population of people who had been given a diagnosis of a ‘personality disorder’ were not downgraded. If the study did not primarily investigate a population and intervention of interest but included this as a subgroup or secondary question, or if the acuity of the service was in any doubt, then the rating was downgraded.

4.        Imprecision: A judgement was made based on whether studies included a power calculation to justify sample size.

5.        Publication bias: We considered whether any included studies had reported non-significant or null results and whether any study protocols or unpublished studies had been found in our search. If the only studies identified as eligible in the search reported positive results or any protocols for unpublished studies were found then the rating was downgraded (if not already at lowest rating).

For contributing studies listed below only the first author is shown.

GRADE scoring results:

Hospital admission:

Outcomes and contributing studies:

Symptomatic improvement: Unger, Giese, Gebhardt, Uhlmann, Yoshimatsu, Branjerdporn

| Model: Hospital admission  Outcome: Symptomatic improvement  Result: Very low certainty  Contributing studies: Uhlmann (quasi-experimental study), Unger, Giese, Gebhardt, Yoshimatsu, Branjerdporn (all cohort studies with pre-post outcomes)  Starting rating: low certainty | | |
| --- | --- | --- |
| Domain | Assessment | Outcome |
| Study quality/risk of bias | Serious limitation:– only one controlled study (quasi-experimental), which was rated as raising ‘serious concerns’ on Robins-I. | Downgraded to very low certainty |
| Inconsistency | Good consistency:– studies were consistent in their results in that all studies reported improvements in symptom scores from admission to discharge. The two studies that used the same measure (BDI) showed very similar scores.  Some consistency also in comparison with other diagnostic groups. | No change |
| Indirectness | Serious indirectness:– In three studies populations also had co-morbid affective disorder and were receiving treatment for this.  Only one study investigated the effect of a model of hospitalisation in crisis specifically for people with a personality disorder diagnosis. | No change as rating already very low and no justification to upgrade |
| Precision | Concerns about imprecision: sample sizes ranged from n=9 to n=68 participants. No sample size calculation for quasi-experimental study.  Sample sizes of n=31 and n=32 in each group likely too low for adequate power considering trials of similar interventions required bigger sample sizes. | No change as rating already very low and no justification to upgrade |
| Publication bias | Concerns about publication bias: Small number of studies.  One study assessed by ROBINS-I assessed as serious risk of bias in selection of reported results.  One study reported non-significant results.  No protocols from unpublished studies found in search strategy. | No change as rating already very low and no justification to upgrade |

| Model: Hospital admission  Outcome: Adaptive functioning  Result: Very low certainty  Contributing studies: Mellsop (cohort study with pre-post outcomes)  Starting rating: low certainty | | |
| --- | --- | --- |
| Domain | Assessment | Outcome |
| Study quality/risk of bias | Serious limitation: only one study and this was rated at high risk of bias. | Downgraded to very low quality |
| Inconsistency | Unable to assess consistency as only one study was available. | No change as rating already very low |
| Indirectness | Concern about indirectness: study was conducted in a general population with mixed diagnoses. | No change as rating already very low |
| Precision | One study with sample size of n=35. | No change as rating already very low |
| Publication bias | Only one small study available. No protocols from unpublished studies found in search strategy. | No change as rating already very low |

Brief admission:

Outcomes and contributing studies:

Symptomatic measures: 2 studies: Barbato and Eckerstrom (both pre-post)

Service use: 4 studies: Koekkek, Van Kessel, Westling, Nehls

NSSI and suicide attempts: 1 study: Westling

Health related QoL: 1 study- Eckerstrom

Therapeutic alliance: 1 study- Koekkoek

| Model: brief admission  Outcome: Service use  Result: Moderate certainty  Contributing studies: Westling (RCT)- compared to TAU, Van Kessel (non- randomised controlled trial)- compared to TAU, Koekkek (quasi-experimental study), Nehls (cohort study with pre-post measures over time) | | |
| --- | --- | --- |
| Study quality/risk of bias | Moderate/serious limitation: Only one RCT, which raised‘some concerns’ on risk of bias assessment; one non-randomised controlled study, which raised concerns about moderate risk of bias. Remaining studies at high risk of bias. | Downgrade certainty to moderate |
| Inconsistency | Consistency in results between RCT and quasi-experimental study; both found within-group reductions in days in hospital but no between-group difference. | No change to certainty - remains moderate |
| Indirectness | RCT and quasi-experimental study both directly investigated population and intervention of interest. | No change to certainty - remains moderate |
| Precision | One study with adequate sample size: the one RCT available reported that sample size was based on a power calculation (n=125)  Other studies: quasi- experimental study sample size n=21.  One study with very small sample size. | No change to certainty - remains moderate |
| Publication bias | RCT was– noted to raise ‘some concerns’ in selective reporting of outcomes but did report non-significant results.  Non- randomised study - low risk of bias.  No smaller studies with null results.  No protocols found for unpublished studies in search strategy. | No change to certainty - remains moderate |

| Model: brief admission  Outcome: Symptomatic measures  Result: Very low certainty  Contributing studies: Barbato (cohort study using pre-post outcomes) and Eckerstrom (cohort study using pre-post outcomes)  Starting rating: low certainty | | |
| --- | --- | --- |
| Study quality/risk of bias | Serious limitation: Two studies at high risk of bias. No controlled studies available. | Downgrade certainty to very low certainty |
| Inconsistency | Consistency in results between studies: both showed an improvement in measures of psychiatric symptoms at p<0.001 | No change to certainty - remains very low |
| Indirectness | Both investigated an intervention of interest. One investigated a population of interest and one investigated a subgroup. | No change to certainty - remains very low |
| Precision | Sample sizes n=36 and n=63.  Both studies reported a pre-post difference at p<0.001 | No change to certainty - remains very low. |
| Publication bias | Both were small studies. No small studies reporting a null result. No protocols from unpublished studies found during search. | No change to certainty - remains very low |

| Model: brief admission  Outcome: NSSI and suicide attempt  Result: moderate certainty  Contributing studies: Westling (RCT)  Starting rating: high certainty | | |
| --- | --- | --- |
| Study quality/risk of bias | The one study included was an RCT and raised ‘some concerns’ on risk of bias assessment | No change to certainty - remains high |
| Inconsistency | Only one contributing study so unable to assess consistency. | No change to certainty - remains high |
| Indirectness | The one study included investigated an intervention of interest in the population of interest. | No change to certainty - remains high |
| Precision | The one study included was informed by a power calculation (n=125) | Downgrade certainty to moderate |
| Publication bias | The one study included reported a null result | No change to certainty - remains moderate |
| Upgrading criteria:  Large effects  Dose response  Plausible residual bias | Not upgraded. |  |

| Model: brief admission  Outcome: Health related QoL  Result: Very low certainty  Contributing studies: Eckerstrom (cohort study reporting pre-post outcomes over time)  Starting rating: low certainty | | |
| --- | --- | --- |
| Study quality/risk of bias | The one study included was considered as at high risk of bias as no control arm. | Downgrade certainty to very low |
| Inconsistency | Unable to assess consistency as only one study available. | No change to certainty - remains very low |
| Indirectness | The one study included investigated an intervention of interest in the population of interest | No change to certainty - remains very low |
| Precision | The one study included had a asample size of n=63, which reported change at p<0.001. | No change to certainty - remains very low |
| Publication bias | The one study included reported a positive finding, which may suggest publication bias. | No change to certainty as already very low |
| Upgrading criteria:  Large effects  Dose response  Plausible residual bias | Not upgraded as null result from RCT, no evidence of dose response, no plausible residual bias. |  |

| Model: brief admission  Outcome: Therapeutic alliance  Result: Very low certainty  Contributing studies: Koekkek (cohort study using pre post outcomes reporting change over time)  Starting rating: low certainty | | |
| --- | --- | --- |
| Study quality/risk of bias | The one study included was considered at high risk of bias. | Downgrade certainty to very low |
| Inconsistency | Unable to assess consistency as only one study was available. | No change to certainty - remains very low |
| Indirectness | The one study included investigated an intervention of interest in the population of interest | No change to certainty - remains very low |
| Precision | The one study included had a small sample size of n= 11 | No change to certainty - remains very low |
| Publication bias | The one study included reported a positive finding, which may suggest publication bias. | No change to certainty - remains very low |
| Upgrading criteria:  Large effects  Dose response  Plausible residual bias | Not upgraded. |  |

Acute day units

Outcomes:

Symptomatic improvement: Savard, Zimmerman, Lariviere, Yen, Vasquez-Bourgon

Social participation: Lariviere

Patient satisfaction: Zimmerman

| Model: Acute day units  Outcome: Symptomatic improvement  Result: Very low certainty  Contributing studies: Zimmerman (quasi-experimental study), Savard, Lariviere, Yen, Vasquez-Bourgon (cohort study reporting pre-post outcomes)  Starting rating: low certainty | | |
| --- | --- | --- |
| Domain | Assessment | Outcome |
| Study quality/risk of bias | The one controlled study included was considered to have a serious risk of bias on the Robins-I.  Remaining studies had no control group and reported pre-post outcomes, so were rated as high risk of bias?? | Downgraded to very low certainty |
| Inconsistency | All studies reporting symptom measures reported statistically significant improvements, although some heterogeneity in effect size (Cohen’s D). | No change to certainty - remains very low |
| Indirectness | In two studies the population sampled was of people with a ‘personality disorder’ diagnosis; theremainder reporting findings of a subgroup from mixed populations.  No concerns about directness of interventions. | No change to certainty - remains very low |
| Precision | Sample sizes ranged from n=20 to n=270  Confidence intervals for Cohen’s D fairly narrow. | No change to certainty - remains very low |
| Publication bias | Small number of studies.  The one controlled study included reported a null result.  No protocols found for unpublished studies in search strategy. | No change to certainty - remains very low |
| Criteria for upgrading | No evidence of large effects, dose response or opposing plausible residual bias and confounding. | No change to certainty - remains very low |

| Model: Acute day units  Outcome: Social participation  Result: Very low certainty  Contributing studies: Lariviere (cohort study reporting pre-post outcomes)  Starting rating: low certainty | | |
| --- | --- | --- |
| Domain | Assessment | Outcome |
| Study quality/risk of bias | The one study included had no control group and was considered to have ahigh risk of bias | Downgraded to very low certainty |
| Inconsistency | Only one study included so could not assess consistency. | No change to certainty as already very low |
| Indirectness | The one study included investigated an intervention of interest in the population of interest, although the acuteness of cases treated by the day hospital was unclear. | No change to certainty - remains very low |
| Precision | Concern about imprecision: sample size of those with a personality disorder diagnosis was n=20, but did detect a change at p<0.001. | No change to certainty as already very low |
| Publication bias | As only one study was identified, this could suggest publication bias. | No change to certainty - remains very low |
| Criteria for upgrading | No evidence of large effects, dose response or opposing plausible residual bias and confounding. | No change to certainty - remains very low |

| Model: Acute day units  Outcome: Patient satisfaction  Result: Very low certainty  Contributing studies: Zimmerman (quasi-experimental study)  Starting rating: low certainty | | |
| --- | --- | --- |
| Domain | Assessment | Outcome |
| Study quality/risk of bias | The one study included was considered to have a high risk of bias. | Downgraded to very low certainty |
| Inconsistency | Only one study included so could not assess consistency. | No change to certainty as already very low |
| Indirectness | The one study included investigated an intervention of interest in the population of interest. | No change to certainty - remains very low |
| Precision | The one study included investigated had a sample size of n=182. No confidence intervals were reported for an effect size relating to this outcome. | No change to certainty as already very low |
| Publication bias | As only one study was identified, this could suggest publication bias. | No change to certainty - remains very low |
| Criteria for upgrading | No evidence of large effects, dose response or opposing plausible residual bias and confounding. | No change to certainty - remains very low |

Model: Outpatient-based psychotherapies/psychosocial interventions:

| Model: Outpatient-based psychotherapies  Outcome: Hospitalisation  Contributing studies: Andreoli (RCT), Grenyer (cluster RCT)  Final rating: moderate certainty  Starting rating: high certainty | | |
| --- | --- | --- |
| Domain | Assessment | Outcome |
| Study quality/risk of bias | The one RCT was rated as raising 'some concerns’ about risk of bias assessment as was the one cluster RCT . | Certainty downgraded to moderate as only 2 studies |
| Inconsistency | Two randomised studies reported consistent findings for service use. | No change to certainty- remains moderate |
| Indirectness | The two randomised studies reported included interventions of interest in the population of interest and both compared intervention to TAU. | No change to certainty - remains moderate |
| Precision | The RCT included a power calculation (sample of n=170).  The cluster RCT did not include a power calculation but did appear to include an adequate number of participants (n=642) to detect a difference in rates of hospitalisation. | No change to certainty - remains moderate |
| Publication bias | The positive findings of the two studies included for this outcome The RCT and cluster RCT were rated as at low risk of bias, which suggests that the positive findings may not reflect selective reporting of the results. .  The cluster RCT also reported some negative results .  No protocols were found for unpublished studies in search strategy. | No change to certainty - remains moderate |
| Criteria for upgrading | No evidence of large effects, dose response or opposing plausible residual bias and confounding | No change to certainty - remains moderate |

| Model: Outpatient based psychotherapies  Outcome: Suicide attempt or ‘suicidal relapse’  Contributing studies: Andreoli (RCT)  Final rating: moderate certainty  Starting rating: high certainty | | |
| --- | --- | --- |
| Domain | Assessment | Outcome |
| Study quality/risk of bias | The one RCT included was rated as at low risk of bias. | No change to certainty - remains high |
| Inconsistency | Only one study included so could not assess consistency. | Certainty downgraded to moderate |
| Indirectness | One study included investigated an intervention of interest in the population of interest | No change to certainty - remains moderate |
| Precision | The one RCT included a power calculation (sample of n=170) | No change to certainty - remains moderate |
| Publication bias | The one RCT included was rated as at low risk of bias, which suggests that the positive findings may not reflect selective reporting of the results. . The  No protocols found for unpublished studies in search strategy. | No change to certainty - remains moderate |
| Criteria for upgrading | No evidence of large effects, dose response or opposing plausible residual bias and confounding. | No change to certainty - remains moderate. |

| Model: Outpatient based psychotherapies  Outcome: Symptomatic improvement  Contributing studies: Huxley, McQuillian, Pavan (all cohort studies reporting pre-post outcomes over time)  Final rating: Very low certainty  Starting rating: low certainty | | |
| --- | --- | --- |
| Domain | Assessment | Outcome |
| Study quality/risk of bias | All were studies without comparison groups and reporting pre-post outcomes, which were all considered as at high risk of bias. | Certainty downgraded to very low |
| Inconsistency | The pre-post studies included reported improvement in symptoms, finding consistent improvements. | No change to certainty - remains very low |
| Indirectness | The pre-post studies included investigated interventions of interest in the population of interest . | No change to certainty - remains very low |
| Precision | Sample sizes were n=22, n=87, n=67.  Studies reported results at p<0.001 | No change to certainty - remains very low |
| Publication bias | The small number of studies reporting positive results could suggest publication bias.  No protocols found for unpublished studies in search strategy. | No change to certainty - remains very low |
| Criteria for upgrading | No evidence of large effects, dose response or opposing plausible residual bias and confounding. | No change to certainty - remains very low |

Model: psychotherapies or psychosocial interventions delivered in emergency departments (ED) or psychiatric emergency services (PES)

| Model: Psychotherapies delivered in ED/PES  Outcome: Repeat suicide attempt  Contributing studies: Berrino (quasi-experimental study)  Starting rating: low certainty  Final rating: very low certainty | | |
| --- | --- | --- |
| Domain | Assessment | Outcome |
| Study quality/risk of bias | The one study included was considered as at serious risk of bias. | Certainty downgraded to very low |
| Inconsistency | Only one study included so could not assess consistency. | No change to certainty - remains very low |
| Indirectness | The one study included investigated an intervention of interest in the population of interest. | No change to certainty - remains very low |
| Precision | Sample size of n=200; did not report confidence intervals. | No change to certainty- remains very low |
| Publication bias | The one study included study reported a positive effect.  One protocol was found (dated 3^rd^ March 2021) during search for an ongoing study, but has not yet reported findings. | No change to certainty as already very low |
| Criteria for upgrading | No evidence of large effects, dose response or opposing plausible residual bias and confounding. | No change to certainty - remains very low |

| Model: Psychotherapies delivered in ED/PES  Outcome: Hospitalisation  Contributing studies: Berrino, Damsa 2003 and Damsa 2005 (both quasi-experimental studies)  Starting rating: low certainty  Final rating: very low certainty | | |
| --- | --- | --- |
| Domain | Assessment | Outcome |
| Study quality/risk of bias | All included studies considered at serious risk of bias on Robins-I. | Certainty downgraded to very low |
| Inconsistency | All included studies showed a consistent direction of effects. | No change to certainty - remains very low |
| Indirectness | All included studies investigated interventions of interest in a population of interest. | No change to certainty - remains very low |
| Precision | Sample sizes of n=507, n=190. N=200. | No change to certainty - remains very low |
| Publication bias | Small number of studies, all reporting positive results, which could suggest publication bias. | No change to certainty as already very low |
| Criteria for upgrading | No evidence of large effects, dose response or opposing plausible residual bias and confounding. | No change to certainty - remains very low |

| Model: Psychotherapies delivered in ED/PES  Outcome: Symptomatic improvement  Contributing studies: Breslow (cohort study with pre-post outcomes), Laddis (quasi-experimental study)  Starting rating: low certainty  Final rating: very low certainty | | |
| --- | --- | --- |
| Domain | Assessment | Outcome |
| Study quality/risk of bias | The quasi-experimental study was considered to be at serious risk of bias on Robins-I.  The cohort study did not have acontrol group. | Certainty downgraded to very low |
| Inconsistency | The two studies showed a consistent direction of effects and found within-group changes over time. | No change to certainty - remains very low |
| Indirectness | The two studies investigated interventions of interest in a population of interest. | No change to certainty - remains very low |
| Precision | Sample sizes were n=69 and n=58. | No change to certainty - remains very low |
| Publication bias | Small number of studies, all reporting positive results, which could suggest publication bias. | No change to certainty as already very low |
| Criteria for upgrading | No evidence of large effects, dose response or opposing plausible residual bias and confounding. | No change to certainty - remains very low |

Model: Psychotherapy groups delivered on inpatient wards

| Model: psychotherapy groups on inpatient wards  Outcome: Symptomatic improvement including distress tolerance  Contributing studies: Springer (RCT), Booth (cohort study with pre-post outcomes)  Starting rating: high certainty  Final rating: low certainty | | |
| --- | --- | --- |
| Domain | Assessment | Outcome |
| Study quality/risk of bias | One RCT rated as at high risk of bias on RoB 2; one cohort rated as at high risk of bias with no control group. | Certainty downgraded to moderate |
| Inconsistency | Both studies showed improvement in scores over time but the randomised study found no between-group differences. | No change to certainty- remains moderate |
| Indirectness | Both studies investigated an intervention of interest in the population of interest. | No change to certainty- remains low |
| Precision | Sample size n=31 for the RCT, and authors reported that trial was likely underpowered.  Sample size n=70 for the pre-post study . | Certainty downgraded to low |
| Publication bias | RCT reported a null result.  Small number of overall studies. No protocols found for unpublished studies in search strategy. | No change to certainty- remains low |
| Criteria for upgrading | No evidence of large effect size, dose response or opposing plausible residual bias and confounding. | No change to certainty- remains low |

| Model: psychotherapy groups on inpatient wards  Outcome: Deliberate self-harm  Contributing studies: Booth (cohort study with pre-post outcomes)  Starting rating: low certainty  Final rating: very low certainty | | |
| --- | --- | --- |
| Domain | Assessment | Outcome |
| Study quality/risk of bias | One study without a control group reported pre-post outcomes and was rated ashigh risk of bias. | Certainty downgraded to very low |
| Inconsistency | Unable to assess consistency as only one study included. | No change to certainty as already very low |
| Indirectness | The one study included investigated an intervention of interest in a population of interest. | No change to certainty- remains very low |
| Precision | Sample size n=70; reported findings at p=0.01; sample size likely adequate for the outcome. | No change to certainty- remains very low |
| Publication bias | Only one study found with positive findings.  No protocols found for unpublished studies in search strategy. | No change to certainty as already very low |
| Criteria for upgrading | No evidence of large effect size, dose response or opposing plausible residual bias and confounding. | No change to certainty- remains very low |

| Model: Joint crisis plans  Outcome: self-harm and symptomatic measures  Contributing studies: Borschmann (pilot RCT)  Starting rating: high certainty  Final rating: low certainty | | |
| --- | --- | --- |
| Domain | Assessment | Outcome |
| Study quality/risk of bias | Only one study found: e pilot RCT with ‘some concerns about risk of bias’. | Certainty downgraded medium |
| Inconsistency | Unable to assess consistency as only one study included. | No change |
| Indirectness | The onestudy included investigated an intervention of interest in a population of interest. | No change |
| Precision | Pilot RCT and so sample size (n=88 likely underpowered to detect effect. | Certainty downgraded to low |
| Publication bias | Only one study found and had negative findings.  No protocols found for unpublished studies in search strategy. | No change |
| Criteria for upgrading | No evidence of large effect size, dose response or opposing plausible residual bias and confounding. | No change |

| Model: Early Intervention Service  Outcome: Symptomatic improvement and social functioning  Contributing studies: Tyrer (RCT)  Starting rating: high certainty  Final rating: | | |
| --- | --- | --- |
| Domain | Assessment | Outcome |
| Study quality/risk of bias | Only one study found: RCT rated as at high risk of bias. | Certainty downgraded medium |
| Inconsistency | Unable to assess consistency as only one study included. | No change |
| Indirectness | The one study included investigated an intervention of interest in a population of interest. | No change |
| Precision | Small sample size as population of interest was a subgroup of the overall trial, therefore likely not powered for this outcome. | Certainty downgraded to low |
| Publication bias | Only one study found and reported mixture of positive and negative results  No protocols found for unpublished studies in search strategy. | No change |
| Criteria for upgrading | No evidence of large effect size, dose response or opposing plausible residual bias and confounding. | No change |

Legend: ED = emergency department; GRADE = Grading of Recommendations, Assessment, Development, and Evaluations; NSSI = non-suicidal self-injury; PES = psychiatric emergency services; RCT = randomised controlled trial.
