# Supplementary material for: Crisis and acute mental health care for people who have been given a diagnosis of a ‘personality disorder’: a systematic review
Source: BMC Psychiatry. 2023 Oct 5;23:720. doi: 10.1186/s12888-023-05119-7 (PMC10552436; doi:10.1186/s12888-023-05119-7)
Supplement: Supplementary file 4 — Additional file 4: Supplementary File 4 (S4). Illustrative examples of quality assessment tools (Rob2 and Robins-I). [file 12888_2023_5119_MOESM4_ESM.docx]

Supplementary File 4 (S4): Illustrative examples of quality assessment tools (Rob2 and Robins-I)

Revised Cochrane risk-of-bias tool for randomized trials (RoB 2)

TEMPLATE FOR COMPLETION

Edited by Julian PT Higgins, Jelena Savović, Matthew J Page, Jonathan AC Sterne
on behalf of the RoB2 Development Group

**Version of 22 August 2019**

The development of the RoB 2 tool was supported by the MRC Network of Hubs for Trials Methodology Research (MR/L004933/2- N61), with the support of the host MRC ConDuCT-II Hub (Collaboration and innovation for Difficult and Complex randomised controlled Trials In Invasive procedures - MR/K025643/1), by MRC research grant MR/M025209/1, and by a grant from The Cochrane Collaboration.


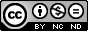


This work is licensed under a [Creative Commons Attribution-NonCommercial-NoDerivatives 4.0 International License](http://creativecommons.org/licenses/by-nc-nd/4.0/).

| **Study details**   \| **Reference** \| Disappointed Love and suicide: a randomised controlled trial of abandonment psychotherapy among borderline patients  Andreoli et al Journal of Personality Disorders 30(2) 271-287, 2016 \| \| --- \| --- \|   **Study design**   \| X \| Individually-randomized parallel-group trial \| \| --- \| --- \| \| □ \| Cluster-randomized parallel-group trial \| \| □ \| Individually randomized cross-over (or other matched) trial \|   **For the purposes of this assessment, the interventions being compared are defined as**   \| Experimental: \| Abandonment psychotherapy \| Comparator: \| TAU \| \| --- \| --- \| --- \| --- \|  \| **Specify which outcome is being assessed for risk of bias** \| Suicidal relapse (attempt or completed) \| \| --- \| --- \| \|  \|  \|  \| **Specify the numerical result being assessed.** In case of multiple alternative analyses being presented, specify the numeric result (e.g. RR = 1.52 (95% CI 0.83 to 2.77) and/or a reference (e.g. to a table, figure or paragraph) that uniquely defines the result being assessed. \| Survival time to suicidal relapse: APP vs TAU pearon chi2=8.09 df=1 p=0.004 \| \| --- \| --- \| \|  \|  \|   **Is the review team’s aim for this result…?**   \| 🞨■ \| to assess the effect of *assignment to intervention* (the ‘intention-to-treat’ effect) \| \| --- \| --- \| \| □ \| to assess the effect of *adhering to intervention* (the ‘per-protocol’ effect) \|   **If the aim is to assess the effect of *adhering to intervention***, select the deviations from intended intervention that should be addressed (at least one must be checked):  □ occurrence of non-protocol interventions  □ failures in implementing the intervention that could have affected the outcome  □ non-adherence to their assigned intervention by trial participants  **Which of the following sources were obtained to help inform the risk-of-bias assessment? (tick as many as apply)**  ◾ Journal article(s) with results of the trial  □ Trial protocol **nb. Protocol not publicly available**  □ Statistical analysis plan (SAP)  □ Non-commercial trial registry record (e.g. ClinicalTrials.gov record)  □ Company-owned trial registry record (e.g. GSK Clinical Study Register record)  □ “Grey literature” (e.g. unpublished thesis)  □ Conference abstract(s) about the trial  □ Regulatory document (e.g. Clinical Study Report, Drug Approval Package)  □ Research ethics application  □ Grant database summary (e.g. NIH RePORTER or Research Councils UK Gateway to Research)  □ Personal communication with trialist  □ Personal communication with the sponsor |
| --- | --- | --- | --- | --- | --- | --- | --- | --- | --- | --- | --- | --- | --- | --- | --- | --- | --- | --- | --- | --- | --- | --- | --- | --- |

## Risk of bias assessment

Responses underlined in green are potential markers for low risk of bias, and responses in red are potential markers for a risk of bias. Where questions relate only to sign posts to other questions, no formatting is used.

**Domain 1: Risk of bias arising from the randomization process**

| **Signalling questions** | **Comments** | **Response options** |
| --- | --- | --- |
| **1.1 Was the allocation sequence random?** | Yes  Yes | Y / PY / PN / N / NI |
| **1.2 Was the allocation sequence concealed until participants were enrolled and assigned to interventions?** |  | Y / PY / PN / N / NI |
| **1.3 Did baseline differences between intervention groups suggest a problem with the randomization process?** | no | Y / PY / PN / N / NI |
| **Risk-of-bias judgement** | Low risk of bias | Low / High / Some concerns |
| Optional: What is the predicted direction of bias arising from the randomization process? |  | NA / Favours experimental / Favours comparator / Towards null /Away from null / Unpredictable |

Domain 2: Risk of bias due to deviations from the intended interventions (*effect of assignment to intervention*)

| **Signalling questions** | **Comments** | **Response options** |
| --- | --- | --- |
| **2.1. Were participants aware of their assigned intervention during the trial?** | Y  Y | Y / PY / PN / N / NI |
| **2.2. Were carers and people delivering the interventions aware of participants' assigned intervention during the trial?** |  | Y / PY / PN / N / NI |
| **2.3. If Y/PY/NI to 2.1 or 2.2: Were there deviations from the intended intervention that arose because of the trial context?** | N | NA / Y / PY / PN / N / NI |
| **2.4 If Y/PY to 2.3: Were these deviations likely to have affected the outcome?** |  | NA / Y / PY / PN / N / NI |
| **2.5. If Y/PY/NI to 2.4: Were these deviations from intended intervention balanced between groups?** |  | NA / Y / PY / PN / N / NI |
| **2.6 Was an appropriate analysis used to estimate the effect of assignment to intervention?** | Y (ITT) | Y / PY / PN / N / NI |
| **2.7 If N/PN/NI to 2.6: Was there potential for a substantial impact (on the result) of the failure to analyse participants in the group to which they were randomized?** |  | NA / Y / PY / PN / N / NI |
| **Risk-of-bias judgement** | Low risk of bias | Low / High / Some concerns |
| Optional: What is the predicted direction of bias due to deviations from intended interventions? |  | NA / Favours experimental / Favours comparator / Towards null /Away from null / Unpredictable |

Domain 2: Risk of bias due to deviations from the intended interventions (*effect of adhering to intervention*)

| **Signalling questions** | **Comments** | **Response options** |
| --- | --- | --- |
| **2.1. Were participants aware of their assigned intervention during the trial?** |  | Y / PY / PN / N / NI |
| **2.2. Were carers and people delivering the interventions aware of participants' assigned intervention during the trial?** |  | Y / PY / PN / N / NI |
| **2.3. [If applicable:] If Y/PY/NI to 2.1 or 2.2: Were important non-protocol interventions balanced across intervention groups?** |  | NA / Y / PY / PN / N / NI |
| **2.4. [If applicable:] Were there failures in implementing the intervention that could have affected the outcome?** |  | NA / Y / PY / PN / N / NI |
| **2.5. [If applicable:] Was there non-adherence to the assigned intervention regimen that could have affected participants’ outcomes?** |  | NA / Y / PY / PN / N / NI |
| **2.6. If N/PN/NI to 2.3, or Y/PY/NI to 2.4 or 2.5: Was an appropriate analysis used to estimate the effect of adhering to the intervention?** |  | NA / Y / PY / PN / N / NI |
| **Risk-of-bias judgement** |  | Low / High / Some concerns |
| Optional: What is the predicted direction of bias due to deviations from intended interventions? |  | NA / Favours experimental / Favours comparator / Towards null /Away from null / Unpredictable |

Domain 3: Missing outcome data

| **Signalling questions** | **Comments** | **Response options** |
| --- | --- | --- |
| **3.1 Were data for this outcome available for all, or nearly all, participants randomized?** | Y | Y / PY / PN / N / NI |
| **3.2 If N/PN/NI to 3.1: Is there evidence that the result was not biased by missing outcome data?** |  | NA / Y / PY / PN / N |
| **3.3 If N/PN to 3.2: Could missingness in the outcome depend on its true value?** |  | NA / Y / PY / PN / N / NI |
| **3.4 If Y/PY/NI to 3.3: Is it likely that missingness in the outcome depended on its true value?** |  | NA / Y / PY / PN / N / NI |
| **Risk-of-bias judgement** | Low risk of bias (as for suicidal relapse there was complete data) | Low / High / Some concerns |
| Optional: What is the predicted direction of bias due to missing outcome data? |  | NA / Favours experimental / Favours comparator / Towards null /Away from null / Unpredictable |

Domain 4: Risk of bias in measurement of the outcome

| **Signalling questions** | **Comments** | **Response options** |
| --- | --- | --- |
| **4.1 Was the method of measuring the outcome inappropriate?** | N | Y / PY / PN / N / NI |
| **4.2 Could measurement or ascertainment of the outcome have differed between intervention groups?** | N | Y / PY / PN / N / NI |
| **4.3 If N/PN/NI to 4.1 and 4.2: Were outcome assessors aware of the intervention received by study participants?** | N | NA / Y / PY / PN / N / NI |
| **4.4 If Y/PY/NI to 4.3: Could assessment of the outcome have been influenced by knowledge of intervention received?** |  | NA / Y / PY / PN / N / NI |
| **4.5 If Y/PY/NI to 4.4:** **Is it likely that assessment of the outcome was influenced by knowledge of intervention received?** |  | NA / Y / PY / PN / N / NI |
| **Risk-of-bias judgement** | Low risk of bias | Low / High / Some concerns |
| Optional: What is the predicted direction of bias in measurement of the outcome? |  | NA / Favours experimental / Favours comparator / Towards null /Away from null / Unpredictable |

Domain 5: Risk of bias in selection of the reported result

| **Signalling questions** | **Comments** | **Response options** |
| --- | --- | --- |
| **5.1 Were the data that produced this result analysed in accordance with a pre-specified analysis plan that was finalized before unblinded outcome data were available for analysis?** | PY | Y / PY / PN / N / NI |
| **Is the numerical result being assessed likely to have been selected, on the basis of the results, from...** |  |  |
| **5.2. ... multiple eligible outcome measurements (e.g. scales, definitions, time points) within the outcome domain?** | PN | Y / PY / PN / N / NI |
| **5.3 ... multiple eligible analyses of the data?** | Y- did not present the adjusted analysis | Y / PY / PN / N / NI |
| **Risk-of-bias judgement** | Some concerns | Low / High / Some concerns |
| Optional: What is the predicted direction of bias due to selection of the reported result? |  | NA / Favours experimental / Favours comparator / Towards null /Away from null / Unpredictable |

Overall risk of bias

| **Risk-of-bias judgement** | Some Concerns- changed after discrepancy meeting | Low / High / Some concerns |
| --- | --- | --- |
| Optional: What is the overall predicted direction of bias for this outcome? |  | NA / Favours experimental / Favours comparator / Towards null /Away from null / Unpredictable |


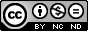


This work is licensed under a [Creative Commons Attribution-NonCommercial-NoDerivatives 4.0 International License](http://creativecommons.org/licenses/by-nc-nd/4.0/).

The Risk Of Bias In Non-randomized Studies – of Interventions (ROBINS-I) assessment tool

(version for cohort-type studies)

**Version 19 September 2016**


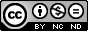


This work is licensed under a [Creative Commons Attribution-NonCommercial-NoDerivatives 4.0 International License](http://creativecommons.org/licenses/by-nc-nd/4.0/).

Study:’ Laddis A. Outcome of crisis intervention for borderline personality disorder and post traumatic stress disorder: a model for modification of the mechanism of disorder in complex post traumatic syndromes. Ann Gen Psychiatry. 2010 Apr 27;9:19. doi: 10.1186/1744-859X-9-19’

## Specify a target randomized trial specific to the study

| Design | Individually randomized |
| --- | --- |
| Participants | Adults admitted to a crisis stabilisation unit who have a diagnosis of a personality disorder or CPTSD |
| Experimental intervention | Psychological intervention according to Cape Cod model |
| Comparator | TAU |

## Is your aim for this study…?

| ■ | to assess the effect of *assignment to* intervention |
| --- | --- |
| □ | to assess the effect of *starting and adhering to* intervention |

## Specify the outcome

Specify which outcome is being assessed for risk of bias (typically from among those earmarked for the Summary of Findings table). Specify whether this is a proposed benefit or harm of intervention.

| Improvement in symptoms according to BPRS and BSI |
| --- |

## Specify the numerical result being assessed

In case of multiple alternative analyses being presented, specify the numeric result (e.g. RR = 1.52 (95% CI 0.83 to 2.77) and/or a reference (e.g. to a table, figure or paragraph) that uniquely defines the result being assessed.

| BPRS: mean score for experimental group 12.9, control 24.7 p<0.001, BSI score at follow up control group mean 84.1 and experimental group 74.2. p=0.314 |
| --- |

## Risk of bias assessment

Responses underlined in green are potential markers for low risk of bias, and responses in red are potential markers for a risk of bias. Where questions relate only to sign posts to other questions, no formatting is used.

|  | **Signalling questions** | **Description** | **Response options** |
| --- | --- | --- | --- |
| **Bias due to confounding** | | | |
|  | 1.1 Is there potential for confounding of the effect of intervention in this study?  **If N/PN to 1.1:** the study can be considered to be at low risk of bias due to confounding and no further signalling questions need be considered | Y | Y / PY / PN / N |
|  | **If Y/PY to 1.1**: determine whether there is a need to assess time-varying confounding: |  |  |
|  | 1.2. Was the analysis based on splitting participants’ follow up time according to intervention received?  **If N/PN**, answer questions relating to baseline confounding (1.4 to 1.6)  **If Y/PY**, go to question 1.3. | N | NA / Y / PY / PN / N / NI |
|  | 1.3. Were intervention discontinuations or switches likely to be related to factors that are prognostic for the outcome?  **If N/PN**, answer questions relating to baseline confounding (1.4 to 1.6)  **If Y/PY**, answer questions relating to both baseline and time-varying confounding (1.7 and 1.8) | N | NA / Y / PY / PN / N / NI |

|  | **Questions relating to baseline confounding only** | | |
| --- | --- | --- | --- |
|  | 1.4. Did the authors use an appropriate analysis method that controlled for all the important confounding domains? | Y | NA / Y / PY / PN / N / NI |
|  | 1.5. **If Y/PY to 1.4**: Were confounding domains that were controlled for measured validly and reliably by the variables available in this study? | Y | NA / Y / PY / PN / N / NI |
|  | 1.6. Did the authors control for any post-intervention variables that could have been affected by the intervention? | N | NA / Y / PY / PN / N / NI |
|  | **Questions relating to baseline and time-varying confounding** | |  |
|  | 1.7. Did the authors use an appropriate analysis method that controlled for all the important confounding domains and for time-varying confounding? |  | NA / Y / PY / PN / N / NI |
|  | 1.8. **If Y/PY to 1.7**: Were confounding domains that were controlled for measured validly and reliably by the variables available in this study? |  | NA / Y / PY / PN / N / NI |
|  | **Risk of bias judgement** | Moderate | Low / Moderate / Serious / Critical / NI |
|  | Optional: What is the predicted direction of bias due to confounding? |  | Favours experimental / Favours comparator / Unpredictable |

| **Bias in selection of participants into the study** | | | |
| --- | --- | --- | --- |
|  | 2.1. Was selection of participants into the study (or into the analysis) based on participant characteristics observed after the start of intervention?  **If N/PN to 2.1:** go to 2.4 | N | Y / PY / PN / N / NI |
|  | 2.2. **If Y/PY to 2.1**: Were the post-intervention variables that influenced selection likely to be associated with intervention?  2.3 **If Y/PY to 2.2**: Were the post-intervention variables that influenced selection likely to be influenced by the outcome or a cause of the outcome? |  | NA / Y / PY / PN / N / NI  NA / Y / PY / PN / N / NI |
|  | 2.4. Do start of follow-up and start of intervention coincide for most participants? | Y | Y / PY / PN / N / NI |
|  | 2.5. **If Y/PY to 2.2 and 2.3, or N/PN to 2.4**: Were adjustment techniques used that are likely to correct for the presence of selection biases? |  | NA / Y / PY / PN / N / NI |
|  | **Risk of bias judgement** | Low | Low / Moderate / Serious / Critical / NI |
|  | Optional: What is the predicted direction of bias due to selection of participants into the study? |  | Favours experimental / Favours comparator / Towards null /Away from null / Unpredictable |

| **Bias in classification of interventions** | | | |
| --- | --- | --- | --- |
|  | 3.1 Were intervention groups clearly defined? | Y | Y / PY / PN / N / NI |
|  | 3.2 Was the information used to define intervention groups recorded at the start of the intervention? | Y | Y / PY / PN / N / NI |
|  | 3.3 Could classification of intervention status have been affected by knowledge of the outcome or risk of the outcome? | N | Y / PY / PN / N / NI |
|  | **Risk of bias judgement** | Low | Low / Moderate / Serious / Critical / NI |
|  | Optional: What is the predicted direction of bias due to classification of interventions? |  | Favours experimental / Favours comparator / Towards null /Away from null / Unpredictable |

| **Bias due to deviations from intended interventions** | | | |
| --- | --- | --- | --- |
|  | **If your aim for this study is to assess the effect of assignment to intervention, answer questions 4.1 and 4.2** | |  |
|  | 4.1. Were there deviations from the intended intervention beyond what would be expected in usual practice? | N | Y / PY / PN / N / NI |
|  | 4.2. **If Y/PY to 4.1**: Were these deviations from intended intervention unbalanced between groups *and* likely to have affected the outcome? |  | NA / Y / PY / PN / N / NI |
|  | **If your aim for this study is to assess the effect of starting and adhering to intervention, answer questions 4.3 to 4.6** | |  |
|  | 4.3. Were important co-interventions balanced across intervention groups? | N | Y / PY / PN / N / NI |
|  | 4.4. Was the intervention implemented successfully for most participants? | Y | Y / PY / PN / N / NI |
|  | 4.5. Did study participants adhere to the assigned intervention regimen? | Y | Y / PY / PN / N / NI |
|  | 4.6. **If N/PN to 4.3, 4.4 or 4.5**: Was an appropriate analysis used to estimate the effect of starting and adhering to the intervention? |  | NA / Y / PY / PN / N / NI |
|  | **Risk of bias judgement** | Moderate (due to differences in co-intervention of medication) | Low / Moderate / Serious / Critical / NI |
|  | Optional: What is the predicted direction of bias due to deviations from the intended interventions? |  | Favours experimental / Favours comparator / Towards null /Away from null / Unpredictable |

| **Bias due to missing data** | | | |
| --- | --- | --- | --- |
|  | 5.1 Were outcome data available for all, or nearly all, participants? | Y | Y / PY / PN / N / NI |
|  | 5.2 Were participants excluded due to missing data on intervention status? | N | Y / PY / PN / N / NI |
|  | 5.3 Were participants excluded due to missing data on other variables needed for the analysis? | N | Y / PY / PN / N / NI |
|  | 5.4 **If PN/N to 5.1, or Y/PY to 5.2 or 5.3**: Are the proportion of participants and reasons for missing data similar across interventions? |  | NA / Y / PY / PN / N / NI |
|  | 5.5 **If PN/N to 5.1, or Y/PY to 5.2 or 5.3**: Is there evidence that results were robust to the presence of missing data? |  | NA / Y / PY / PN / N / NI |
|  | **Risk of bias judgement** | Low | Low / Moderate / Serious / Critical / NI |
|  | Optional: What is the predicted direction of bias due to missing data? |  | Favours experimental / Favours comparator / Towards null /Away from null / Unpredictable |

| **Bias in measurement of outcomes** | | | |
| --- | --- | --- | --- |
|  | 6.1 Could the outcome measure have been influenced by knowledge of the intervention received? | Y | Y / PY / PN / N / NI |
|  | 6.2 Were outcome assessors aware of the intervention received by study participants? | Y | Y / PY / PN / N / NI |
|  | 6.3 Were the methods of outcome assessment comparable across intervention groups? | Y | Y / PY / PN / N / NI |
|  | 6.4 Were any systematic errors in measurement of the outcome related to intervention received? | N | Y / PY / PN / N / NI |
|  | **Risk of bias judgement** | Serious (outcome assessment unblinded and vulnerable to subjectivity) | Low / Moderate / Serious / Critical / NI |
|  | Optional: What is the predicted direction of bias due to measurement of outcomes? |  | Favours experimental / Favours comparator / Towards null /Away from null / Unpredictable |

| **Bias in selection of the reported result** | | | |
| --- | --- | --- | --- |
|  | Is the reported effect estimate likely to be selected, on the basis of the results, from... |  |  |
|  | 7.1. ... multiple outcome *measurements* within the outcome domain? | PN | Y / PY / PN / N / NI |
|  | 7.2 ... multiple *analyses* of the intervention-outcome relationship? | PN | Y / PY / PN / N / NI |
|  | 7.3 ... different *subgroups*? | PN | Y / PY / PN / N / NI |
|  | **Risk of bias judgement** | Low | Low / Moderate / Serious / Critical / NI |
|  | Optional: What is the predicted direction of bias due to selection of the reported result? |  | Favours experimental / Favours comparator / Towards null /Away from null / Unpredictable |

| **Overall bias** | | | |
| --- | --- | --- | --- |
|  | **Risk of bias judgement** | Serious | Low / Moderate / Serious / Critical / NI |
|  | Optional: What is the overall predicted direction of bias for this outcome? |  | Favours experimental / Favours comparator / Towards null /Away from null / Unpredictable |


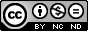


This work is licensed under a [Creative Commons Attribution-NonCommercial-NoDerivatives 4.0 International License](http://creativecommons.org/licenses/by-nc-nd/4.0/).
